# Secondary Ion Mass Spectrometry Imaging of *Dictyostelium discoideum* Aggregation Streams

**DOI:** 10.1371/journal.pone.0099319

**Published:** 2014-06-09

**Authors:** John Daniel DeBord, Donald F. Smith, Christopher R. Anderton, Ron M. A. Heeren, Ljiljana Paša-Tolić, Richard H. Gomer, Francisco A. Fernandez-Lima

**Affiliations:** 1 Department of Chemistry and Biochemistry, Florida International University, Miami, Florida, United States of America; 2 FOM Institute AMOLF, Science Park 104, Amsterdam, The Netherlands; 3 Environmental Molecular Sciences Laboratory, Pacific Northwest National Laboratory, Richland, Washington, United States of America; 4 Department of Biology, Texas A&M University, College Station, Texas, United States of America; University of Dundee, United Kingdom

## Abstract

High resolution imaging mass spectrometry could become a valuable tool for cell and developmental biology, but both, high spatial and mass spectral resolution are needed to enable this. In this report, we employed Bi_3_ bombardment time-of-flight (Bi_3_ ToF-SIMS) and C_60_ bombardment Fourier transform ion cyclotron resonance secondary ion mass spectrometry (C_60_ FTICR-SIMS) to image *Dictyostelium discoideum* aggregation streams. Nearly 300 lipid species were identified from the aggregation streams. High resolution mass spectrometry imaging (FTICR-SIMS) enabled the generation of multiple molecular ion maps at the nominal mass level and provided good coverage for fatty acyls, prenol lipids, and sterol lipids. The comparison of Bi_3_ ToF-SIMS and C_60_ FTICR-SIMS suggested that while the first provides fast, high spatial resolution molecular ion images, the chemical complexity of biological samples warrants the use of high resolution analyzers for accurate ion identification.

## Introduction

The interrogation of biological systems with secondary ion mass spectrometry (SIMS) has seen significant growth over the last decade. [1], [2], [3], [4], [5], [6], [7] This relatively newfound application of a surface technique traditionally limited to the study of inorganic and small molecule analytes is largely derived from the advent of larger, cluster primary ion probes (e.g., C_60_, [8], [9], [10], [11] Ar clusters, [12], [13] and Au nanoparticles [14], [15], [16], [17], [18], [19]) which provide enhanced secondary ion yields of molecular and fragment ions from biological samples. While the use of traditional time of flight (TOF-SIMS) and magnetic sector based methodologies have intrinsic advantages for the *in situ* analysis of surfaces (e.g., speed, sensitivity, dynamic range, depth profiling), the complexity and number of components usually encountered in the analysis of biological systems warrant the coupling of these new sources to high mass accuracy and resolution analytical devices for direct identification of the molecules of interest. [20], [21], [22], [23], [24] In particular, this requirement grows out of the need for improved identification certainty for molecular ions generated from biological samples, which are substantially more complex relative to semiconductor and polymer-based applications, where the number of sample components is limited and the analyte of interest is typically predetermined.

Previous mass spectrometry imaging studies have shown the advantages of correlating spatial information with molecular composition for the study of a variety of biological systems. [25] A common drive has been the search for biological models and better interrogation probes with higher spatial resolution and improved molecular identification. To this end, we used *Dictyostelium discoideum* as a biological model for evaluating the performance of two different mass spectrometry imaging approaches. *D. discoideum* cells are eukaryotic cells that normally live on soil surfaces and eat bacteria. [26], [27] An interesting feature of their biological cycle is that when the cells overgrow their food supply and starve, they aggregate together in dendritic streams to form groups of ∼20,000 cells. The aggregated cells eventually form a fruiting body consisting of a 1–2 mm tall stalk supporting a mass of spore cells which can then be dispersed by the wind to start new colonies. Because soil surfaces are exposed to rain water, the cells can survive and undergo development in water. This feature makes *D. discoideum* a good model for *in situ* mass spectrometry imaging since it does not require the use of cleaning protocols that can potentially compromise the spatial information (e.g., removal of buffer salts and/or media components). In addition, this cell averages 10 µm in size, which is at the frontier of various surface interrogation techniques (e.g., SIMS, DESI and MALDI). [5], [25], [28] Although the lipid composition of *D. discoideum* has been studied at different developmental stages using traditional chromatographic techniques and mass spectrometry, [26], [29], [30], [31], [32] nothing is known about their distribution during chemotaxis and the aggregation process. In this article, we explore the potential for SIMS imaging of unknown biological samples by employing traditional TOF-SIMS and accurate mass determination via FTICR-SIMS for direct molecular ion identification of biological components in *D. discoideum* during aggregation.

## Experimental Method

### Sample Preparation

*D. discoideum* Ax2 cells were grown in shaking culture at 21°C in Formedium HL-5 as previously described. [33] Mid-log cells (1–2×10^6^ cells/ml) were collected by centrifugation at 1,500 x g for 4 minutes, resuspended in PBM (20 mM KH_2_PO_4_, 10 µM CaCl_2_, 1 mM MgCl_2_, pH 6.1), and collected by centrifugation. The resuspension and centrifugation were repeated two more times. The cells were resuspended in PBM to 5×10^6^ cells/ml, and 10 ml of cells was placed in a 125 ml Erlenmeyer flask and shaken at room temperature for 4 hours. The cells were then diluted 1∶6 with PBM, collected by centrifugation, and resuspended in deionized water. The collection and resuspension in deionized water were repeated twice, and the cells were diluted to 9×10^5^ cells/ml. 80 µl droplets of the cells were then spotted onto gold-coated silicon chips (Sigma Aldrich). After allowing cells to settle for 30 minutes, 40 µl of the overlaying water was removed and the chips were placed in a humid box at 21°C. 17 hours later, the chips with aggregating cells were gently drained by touching to a kimwipe, and placed cell-side down on a piece of dry ice. This was covered by a piece of aluminum foil, inverted, and placed in a vacuum chamber. After 12 hours, the dry ice had evaporated and the sample was dessicated. The chips with cells were then stored over a CaCl_2_ desiccant at room temperature.

### Instrumentation

Duplicate *D. discoideum* samples were analyzed in positive ion mode using a ToF SIMS^5^ instrument (ION-TOF, Münster, Germany) and a custom C_60_ FTICR-SIMS. The custom C_60_ FTICR-SIMS instrument (more details in refs [21], [23]) utilizes a 40 keV C_60_ primary ion gun (Ionoptika Ltd., Hampshire, England) that is coupled to a SolariX 9.4T FTICR mass spectrometer (Bruker Daltonics Inc, Billerica, MA). The vacuum pumping scheme of the SolariX cart was modified so that the pressure in the source chamber was reduced to 3×10^−5^ mbar instead of the ∼3 mbar at which it typically operates. The C_60_ FTICR-SIMS images were acquired using 40 keV C_60_^+^ projectiles over a field of view of approximately 4 mm×6 mm with a pixel size of 125 µm and a total primary ion dose of 2.78×10^13^ ions/cm^2^. Spectra were acquired using a broadband excitation over the 100<m/z<1,500 range, with 1.0 s transients collected for each pixel. Transients were zero-filled and Sine-Bell apodized prior to fast Fourier transformation. An ion accumulation time of 0.40 s was used to obtain sufficient S/N in the resulting spectra. In the case of the ToF-SIMS analysis, no modifications were made to the instrument. The analysis was performed by rastering the 25 keV Bi_3_^+^ beam over a 500 µm^2^ field of view with a pixel size of 3.9 µm and a total primary ion dose of 8.16×10^12^ ions/cm^2^.

### Data Analysis

Spectra and images from the Bi_3_ ToF-SIMS analysis were processed using SurfaceLab 6 software (ION-TOF, Münster, Germany). C_60_ FTICR-SIMS images were visualized using FlexImaging software (Bruker Daltonics Inc., Billerica, MA). Peak signals were identified using mMass software [34], [35] from the summed spectrum of all pixels within the region of interest. A signal to noise threshold of 10 was used to generate a peak list containing 2,595 peaks. This peak list was then searched against the LIPID MAPS database (www.lipimaps.org), which contains ∼37,000 entries, using the mMass compound search tool.[34], [35] In addition to the typical protonated ions, sodium and potassium adducts as well as dehydration rearrangement products (-H_2_O) were considered. These assignment criteria returned 293 peaks which could be matched to a lipid ion with better than 5 ppm mass accuracy. All reported ion masses were measured from the total spectrum summed over all image pixels.

## Results and Discussion

**Figure 1:**
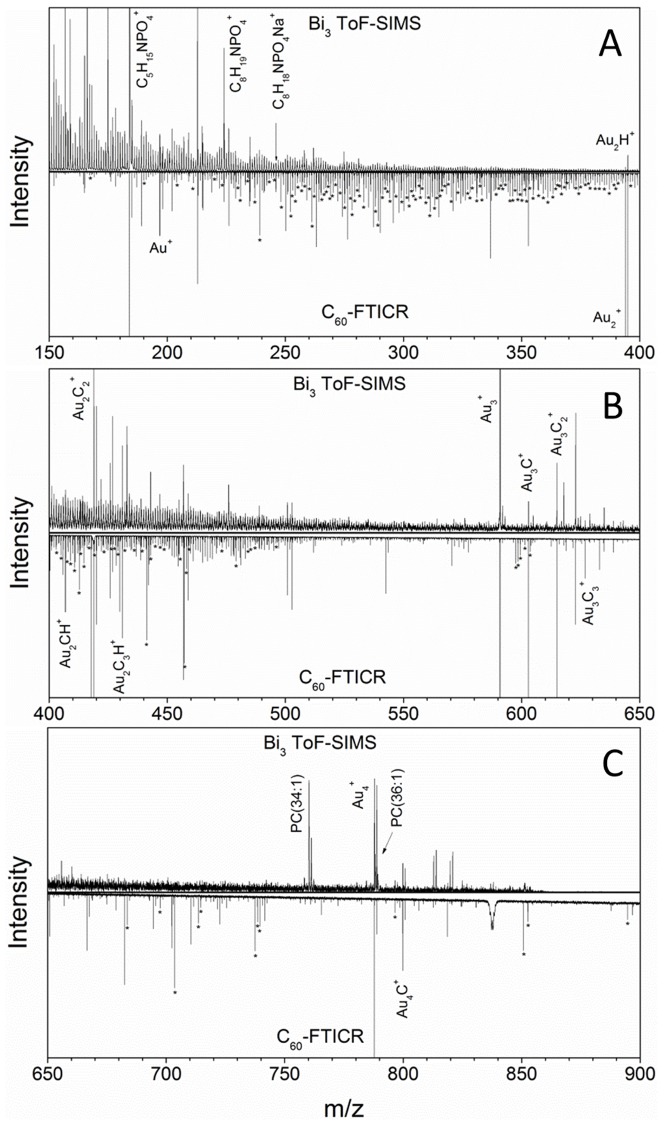
Comparison of Bi_3_ ToF-SIMS and C_60_ FTICR-SIMS spectra. Bi_3_ ToF-SIMS (top of each panel) and C_60_ FTICR-SIMS (inverted in each panel) spectra of aggregating *D. discoideum* cells. Lipid fragments, gold clusters, and gold cluster adduct peaks are labeled with their corresponding molecular formulas while lipid species identified from the LIPID MAPS database are denoted with asterisks (*).

We observed that when *D. discoideum* cells are starved in water, aggregation stream formation begins at about 16 hours, compared to the ∼8 hours when cells are starved in buffer. However, stream formation was not further delayed when cells were starved on a gold surface instead of the usual glass or plastic surfaces used for most work with this organism. Typical mass spectra of aggregating *D. discoideum* cells from Bi_3_ ToF-SIMS and C_60_ FTICR-SIMS are shown in Figure 1. The mass range and ion relative abundances are similar for each instrument and both are characteristic of SIMS analyses of biological targets. That is, the SIMS spectra are dominated by singly charged ions in the 0<m/z<500 range with some larger ion species (500< m/z <1200) present at lower abundance. A common feature between the spectra is the fact that the most intense peaks correspond to gold cluster and gold cluster hydrocarbon adduct ions derived from the gold-coated silicon wafer substrate (as expected, since this constitutes the majority of the surface area within the analyzed region). The gold cluster species were used to internally calibrate the FTICR-SIMS spectrum summed over all pixels to a mass accuracy below 5 ppm. As a figure of merit, a mass resolving power of ∼150,000 (*m*/Δ*m*_50%_) was measured at m/z = 393.9326 (Au_2_^+^ peak), where Δ*m*_50%_ is the magnitude mode spectral peak width at half-maximum peak height. The C_60_ FTICR-SIMS spectrum also shows numerous lipid-specific fragments, with the most abundant being the phosphatidylcholine head group (C_5_H_15_NPO_4_^+^) at m/z 184. A total of 293 peaks in the C_60_ FTICR-SIMS spectrum can be attributed to lipid species. When comparing the Bi_3_ ToF-SIMS and C_60_ FTICR-SIMS spectra, there are some key differences that become apparent. (1) The radio-frequency ion guides and quadrupole (set to transmit m/z 160 and above) used to transfer ions from the source to the ICR cell induce a low mass cutoff as seen by the significant reduction in ion signal below m/z = 200 (relative to the ToF-SIMS spectrum). [36] (2) The greater number of lipid signals detected in the 650<m/z<900 range for C_60_ show that this large cluster projectile is more efficient for generating intact lipid molecular ions than smaller primary ions such as Bi_3_ (as previously noted in ref [37]). It is important to note that the ion fluences used were 2.78×10^13^ ions/cm^2^ and 8.16×10^12^ ions/cm^2^ for the C_60_ and Bi_3_ analysis, respectively. These values are at or slightly above the static limit, meaning that erosion of the sample is expected. According to the reported sputter yields for these projectiles in organic matrices at the similar fluences and kinetic energies, [38], [39], [40] the sampled depths are estimated to be approximately 50 nm and 15 nm for the C_60_^+^ and Bi_3_^+^ analyses, respectively.

**Figure 2:**
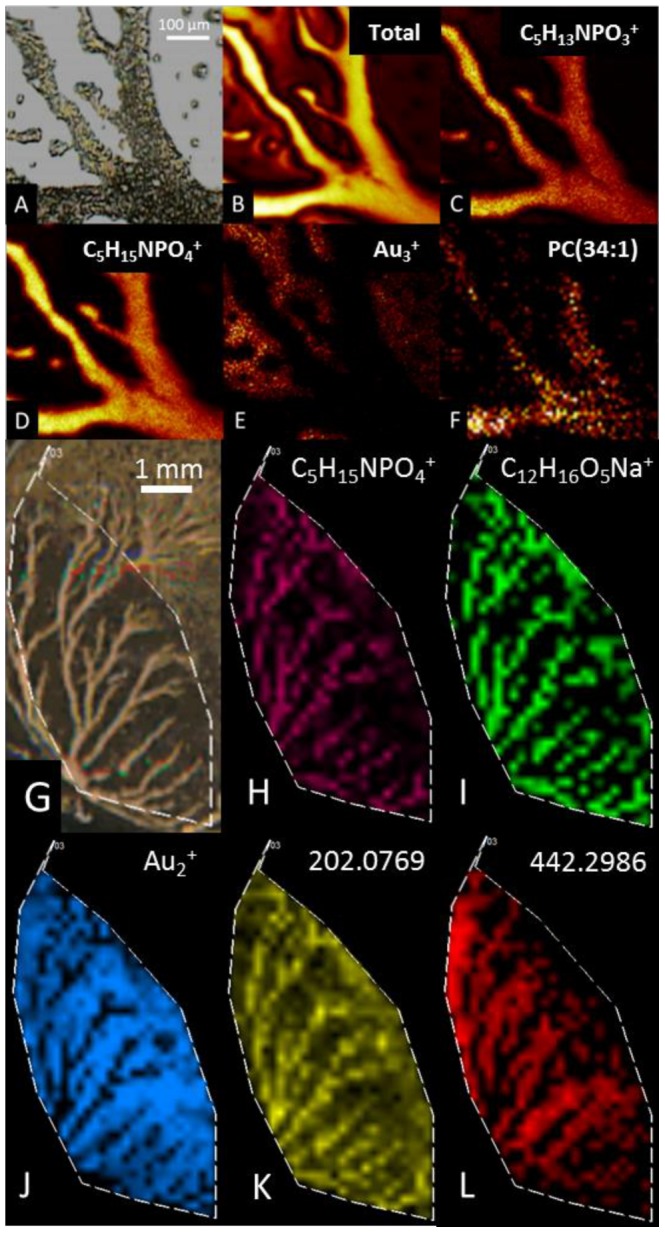
Optical and secondary ion images of *D. discoideum* aggregation streams. (A) Optical and (B–F) ion images of aggregation streams generated by 25 keV Bi_3_ TOF-SIMS analysis. (G) Optical and (H–L) ion images of aggregation streams generated by 40 keV C_60_ FTICR-SIMS analysis.

Figure 2 shows optical and selected ion images from the Bi_3_ ToF-SIMS (Figure 2: B–F) and C_60_ FTICR-SIMS (Figure 2: H–L) spectra. The optical microscopy images (Figure 2: A, G) clearly show the cellular aggregation streams which form branched structures <200 µm in width and a few millimeters in length. The ToF-SIMS total secondary ion image (Figure 2B) shows higher intensity for ions originating from the aggregation streams and lower overall intensity from the gold substrate. C_5_H_13_NPO_3_^+^ (m/z  = 166.1), C_5_H_15_NPO_4_^+^ (m/z  = 184.1), and m/z  = 760.6 give spatial distributions corresponding to the aggregation streams. The C_5_H_13_NPO_3_^+^ and C_5_H_15_NPO_4_^+^ species are head group fragment ions from glycerophosphatidylcholines, which make up ∼25% of all lipids present in *D. discoideum*. [31] The signal at m/z  = 760.6 appears to be a lipid molecular ion due to its co-localization with the aggregations streams, the observed isotopic pattern which contains significant ^13^C contributions, and the presence of another peak at m/z  = 788.6 corresponding to the same molecule with a fatty acyl chain two carbons longer. [41], [42] However, due to the limited mass accuracy afforded by ToF analysis and their absence from the C_60_ FTICR-SIMS spectrum, the precise identities of these supposed lipids was not determined. Viewing the sample as a binary system containing signals from the cellular aggregations and from the substrate, we are also able to show that the Au_3_^+^ ion image represents only the substrate as this signal is not observed from the aggregations streams.

Analogously, molecular ion images can also be obtained from the C_60_ FTICR-SIMS spectra. Two of the mass spectral features (Figure 2: H, I) which display spatial distributions corresponding to the aggregation streams are C_5_H_15_NPO_4_^+^ (m/z = 184.0737, δ = 2.1 ppm) and C_12_H_16_O_5_Na^+^ (m/z = 263.0889, δ = −0.2 ppm). As mentioned above, C_5_H_15_NPO_4_^+^ corresponds to the phosphatidylcholine head group, while according to the LIPID MAPS database, the C_12_H_16_O_5_Na^+^ species corresponds to the heterocyclic fatty acyl 3-carboxy-4-methyl-propyl-2-furanpropanoic acid (LIPID MAPS ID: LMFA01150004), which has previously been detected from human uremic serum as a sodiated ion using SIMS. [43] Ion images for two unidentified peaks from the FTICR spectrum are shown in panels K and L. The image of m/z 202.0769 shows a distribution consistent with the aggregation streams with lower level concentrations between the aggregation streams. The m/z 442.2986 ion is located on the surface in proximity to, but not within the aggregation streams. Such an arrangement may mean this ion corresponds to a metabolite which is secreted from the *D. discoideum* cells. The m/z 202.0769 and 442.2986 ions did not return lipid matches within the 5 ppm mass accuracy threshold, suggesting these lipids are not contained in the database, these compounds are not lipids, or the mass errors for these peaks fall outside the applied threshold range. As such, identities for these ions can not be determined from this analysis. As in the ToF-SIMS analysis, a Au-related ion, Au_2_^+^ (m/z 393.9326, δ = −0.1 ppm), can be used to visualize the substrate and not the aggregation streams.

**Figure 3:**
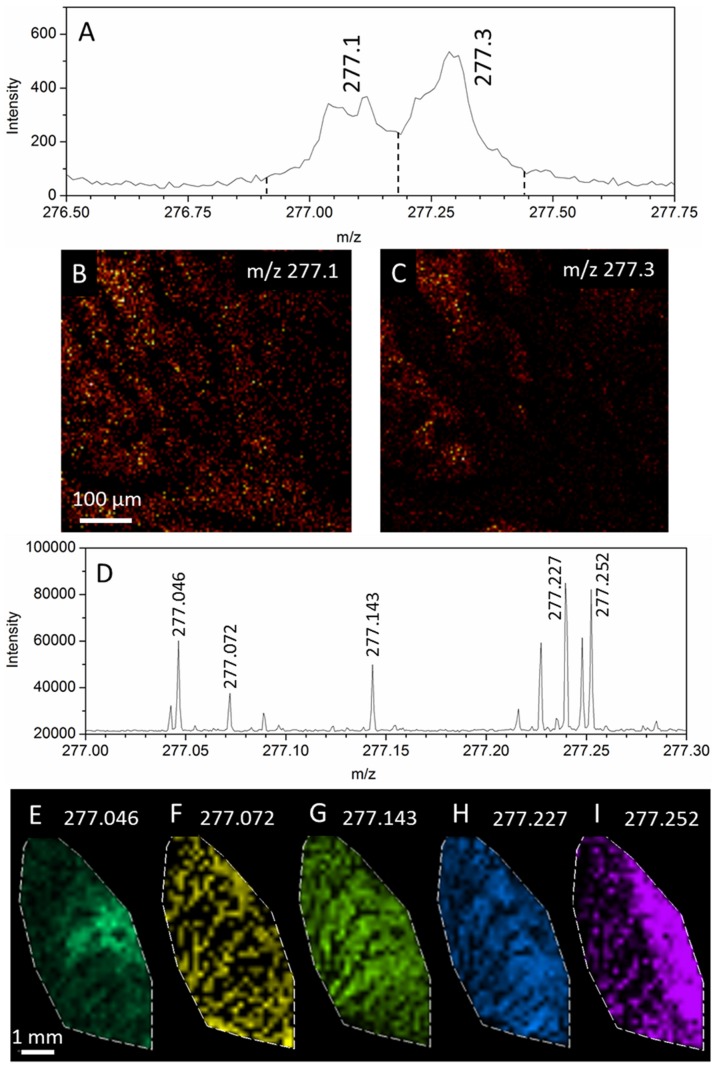
Secondary ion images from within the m/z = 277 nominal mass. (A) Bi_3_ ToF-SIMS and (D) C_60_ FTICR-SIMS spectra excerpts showing multiple peaks within the 277 nominal mass. (B,C) Bi_3_ ToF-SIMS ion images obtained from the first “peak” and second “peak” within the 277 nominal mass. (E–I) C_60_ FTICR-SIMS ion images generated for the corresponding peaks in D with a m/z bin size of +/− 0.001.

The molecular ion images shown in Figure 2 demonstrate that ions throughout the mass range can be used to display meaningful spatial distributions. Moreover, the mass resolving power of the FTICR-SIMS instrument is most apparent when the true complexity of the sample is revealed. The excerpted mass spectrum (from the sum of all spectra) shown in Figure 3D shows that within the spectrum, there can be upwards of 10 ions within a given nominal mass, and that each of these ions may arise from different regions within the sample. Assuming a composition of carbon, hydrogen, nitrogen, oxygen, and phosphorus and a 5 ppm threshold, we can suggest molecular formulas for the peaks at m/z = 277.046, 277.072, 277.143, 277.227, and 277.252 m/z to be C_12_H_9_N_2_O_6_^+^ or C_10_H_14_O_7_P^+^, C_14_H_13_O_6_^+^, C_16_H_21_O_4_^+^, C_17_H_29_N_2_O^+^, and C_19_H_33_O^+^ respectively. From the ion images it is apparent that two of the ions (277.072 and 277.252) originate from the aggregation streams. As expected, these two ions are the only two which match entries from the LIPID MAPS database. The m/z = 277.072 ion can be identified as Thysanone (LIPID MAPS ID: LMPK13030001, [C_14_H_12_O_6_+H]^+^, δ = −4.9 ppm). [44] The peak at 277.252 has the formula [C_19_H_32_O+H]^+^ (δ = −0.2 ppm) and has 13 possible lipid matches with the same stoichiometry. This list of matches includes a sterol lipid, 4 prenol lipids, and 8 sphingolipids. Analysis with ToF-SIMS (Figure 3A) reveals at least two unresolved peaks within the m/z 277 nominal mass. The selected ion images generated by integrating the left and right halves of the peak cluster (3B,3C) show some differences in spatial distribution, but the unique distributions of the summed peaks are lost due to insufficient mass resolving power. Further attempts to segment the peaks resulted in insufficient counts per window to generate ion images (see Figure S1).

**Figure 4:**
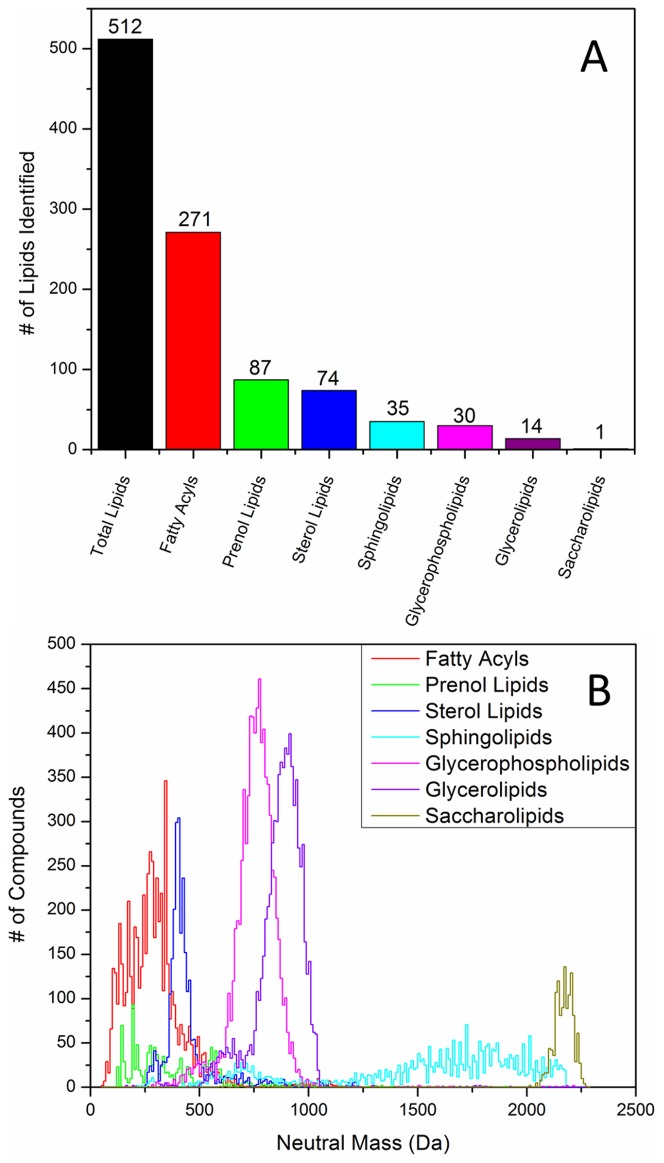
Overlap between the detected lipid classes and the LIPID MAPS database. (A) The number of peaks identified from the C_60_-FTICR-SIMS spectrum by lipid class. (B) Mass distributions for compounds from the LIPID MAPS database organized by lipid class. Each data point represents the number of lipids for a given class binned every 10 mass units.

The search for lipid IDs from the high resolution FTICR-SIMS spectrum against the LIPID MAPS database resulted in 293 hits throughout the spectrum within 5 ppm mass measurement accuracy. Depending on the uniqueness of each detected m/z, peaks can be assigned to a single lipid, any of multiple isomers within a given class, or to any of multiple isomers from multiple lipid classes. A summary plot showing the 512 lipid class assignments for the 293 peaks is provided in Figure 4A, with the detailed list of peak assignments included as Table S1. Figure 4B shows the number of compounds from each lipid class in the LIPID MAPS database binned every 10 Da. The three most commonly detected classes of lipids, the fatty acyls, prenol lipids, and sterol lipids, feature mass distributions which reside almost entirely within the 200–500 Da range. This happens to be the range over which most of the C_60_ FTICR-SIMS signal is observed. As a general trend, as the mass of the compound increases, the probability of ion formation/survival decreases in SIMS analyses. Only one saccharolipid (LMSL05000001, C_18_H_32_O_8_) was detected due to the fact that nearly all saccharolipids in the database reside at >2,000 Da. Most of the detected species, especially the nonpolar and electronegative compounds (like fatty acyls), were detected as sodiated or potassiated ions, with most undergoing dehydration reactions in order to generate positive ions.

Previous reports of the *D. discoideum* lipid profile are almost exclusively limited thin-layer chromatographic measurements of the types and relative abundances of the general lipid classes without regard to the specific lipids present. As an example, Paquet et al. recently reported that neutral, phosphoethanolamine, and phosphocholine lipids constitute over 80% of a total lipid extract from *D. discoideum*. However, variations in ionization probability between lipid classes and a mass dependent detection probability preclude quantitative comparisons of this type from mass spectrometric data. There have also been many reports of the fatty acid profile of *D. discoideum* obtained from hydrolyzed lipid extracts, but these fatty acyls have not been linked back to their parent lipid class. In order to obtain a more detailed lipid profile, the fatty acyls should be detected along with their corresponding head groups. This could be done either by using a solvent prefractionation method to isolate the various lipid class prior to hydrolysis and subsequent GC analysis [45] or by analysis of the original intact molecular ions [24] as was done here. This has been done for the most abundant sphingolipids from *D. discoideum* using liquid chromatography mass spectrometry; [32] however, this analysis was performed in negative ion mode while our MS analyses were acquired in positive ion mode. The author did propose identities for the four most abundant lipids observed in positive ion mode to be PC(36∶4), PC(34∶4), PC(32∶2), and PS(32∶1), but these ions were not observed in the C_60_ FTICR-SIMS spectrum.

Despite the fact that lipid profiling using this approach is biased by the mass range and ionization probability of the desorbed molecules, it does offer a rapid tool for molecular differentiation and cell state classification. A current limitation of this approach (e.g., compared to LC-MS lipid profiling) lies in the inability to differentiate isobaric species. The identity of structural isomers is often important in lipid analysis and efforts have been made to incorporate MS/MS capabilities into SIMS analysis. [46], [47]. The current FTICR-SIMS instrument is also capable of MS/MS measurements, [21] though none were performed during the course of this study. Another limitation of the current prototype lies in the sub-optimal focusing of the C_60_^+^ primary ion beam which has a diameter of ∼75 µm and the lack of ion raster optics which means mechanical stage movement must be used to generate ion images rather than the more precise method of beam rastering. Other groups have shown that C_60_ beams can be focused down to 200 nm and rastered to create images with sub-micron spatial resolution. [48] Such improvements would be necessary for the current instrument to resolve smaller surface features such as lipid distributions within *Dictyostelium* aggregation streams or individual *Dictyostelium* cells.

## Conclusions

Bi_3_ ToF-SIMS and C_60_ FTICR-MS offer complementary information, where the first analysis provides short analysis times and high spatial resolution while the second demonstrates the need for higher mass resolving power when interrogating biological samples. In particular, the use of high mass resolving power in SIMS (e.g., FTICR-SIMS) was shown to be effective for the analysis of a variety of chemical classes with molecular ion masses <1,000 Da (e.g., fatty acyls, prenol lipids, and sterol lipids). Further incorporation of high resolution mass analyzers with high spatial resolution surface probes will permit a better identification of molecular components in biological matrices, a necessary step in the progression towards single cell mass spectrometry imaging.

## Supporting Information

Figure S1 **Bi_3_ ToF-SIMS secondary ion images from within the m/z = 277 nominal mass.** (A–D) Bi_3_ ToF-SIMS selected ion images produced from the (E) segmented peaks within the m/z 277 nominal mass. (F) Optical image and (G) summed image of the full m/z 277 peak.
(TIF)

Table S1 **List of lipids with <5 ppm mass error identified from the LIPID MAPS database.**
(DOCX)
