# Supplementary figures and images for: Secondary Ion Mass Spectrometry Imaging of Dictyostelium discoideum Aggregation Streams
Source: PLoS One. 2014 Jun 9;9(6):e99319. doi: 10.1371/journal.pone.0099319 (PMC4049834; doi:10.1371/journal.pone.0099319)

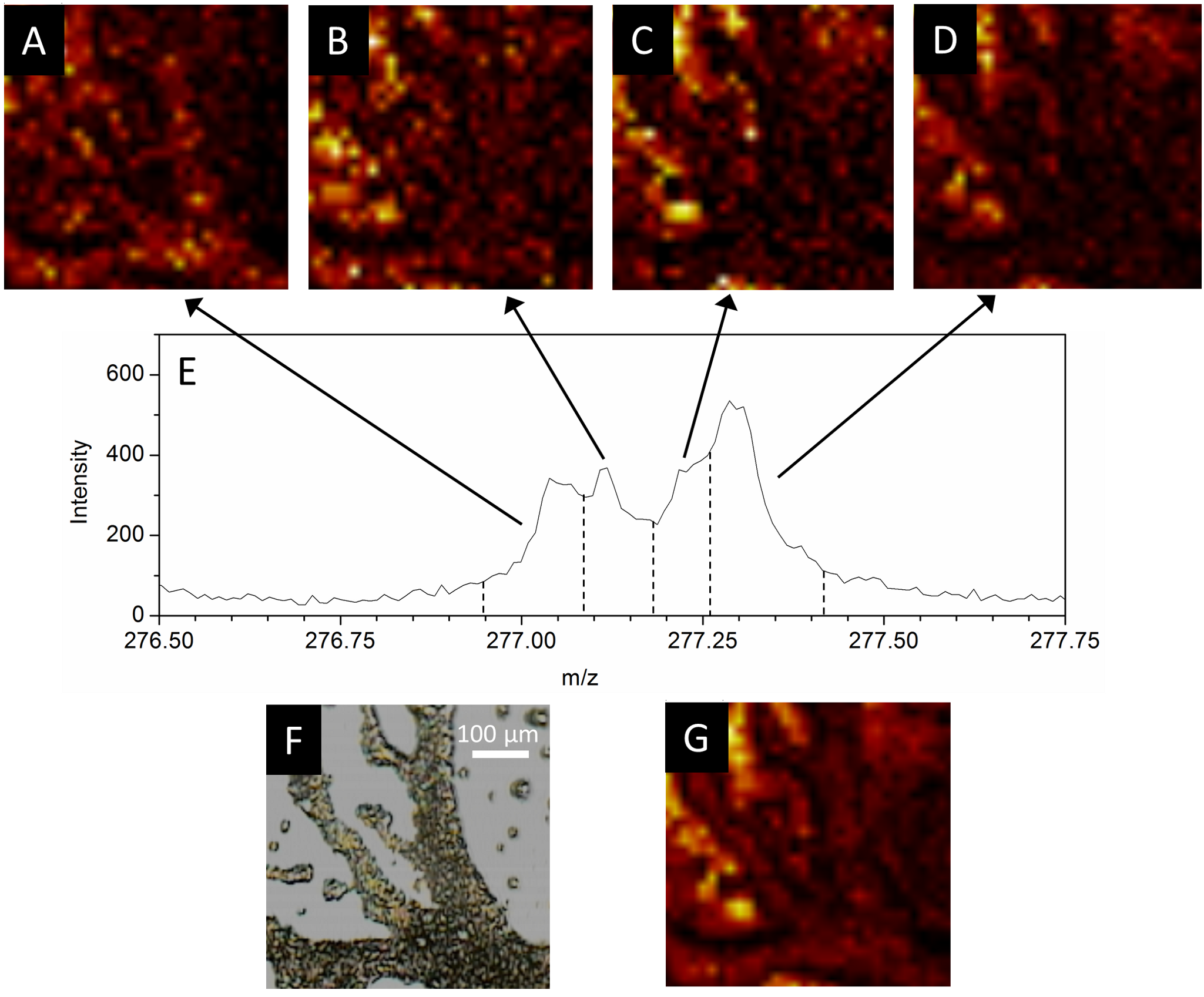

Supplement: Figure S1 — Bi3 ToF-SIMS secondary ion images from within the m/z = 277 nominal mass. (A–D) Bi3 ToF-SIMS selected ion images produced from the (E) segmented peaks within the m/z 277 nominal mass. (F) Optical image and (G) summed image of the full m/z 277 peak. (TIF) [file pone.0099319.s001.tif]
