# Supplementary material for: Secondary Ion Mass Spectrometry Imaging of Dictyostelium discoideum Aggregation Streams
Source: PLoS One. 2014 Jun 9;9(6):e99319. doi: 10.1371/journal.pone.0099319 (PMC4049834; doi:10.1371/journal.pone.0099319)
Supplement: Table S1 — List of lipids with <5 ppm mass error identified from the LIPID MAPS database. (DOCX) [file pone.0099319.s002.docx]

**Table S1.** List of lipids with < 5ppm mass error identified from the LIPID MAPS database.

| \| **Meas.**  **m/z** \| \| --- \| | **Calc. m/z** | **δ**  **(ppm)** | **Rel. Int. (%)** | **Formula** | **Annotation** |
| --- | --- | --- | --- | --- | --- | --- |
| 117.0674 | 117.0675 | -0.8 | 0.18 | C7H12O(Na)(H-2O-1)(H-1) | LMFA05000119; LMFA12000087; LMFA12000084; LMFA12000080; LMFA06000022; LMFA06000023; LMFA06000020; LMFA06000021; LMFA12000104; LMFA12000103; LMFA12000010 ; LMFA12000097;LMFA12000098; LMFA06000019; LMFA12000110 |
| 145.0645 | 145.0648 | -2.3 | 0.09 | C10H8O | LMFA06000062 |
| 145.0645 | 145.0648 | -2.3 | 0.09 | C10H10O2(H-2O-1) | LMPR0102110001 |
| 145.0645 | 145.0648 | -2.3 | 0.09 | C10H10O2(H-2O-1) | LMFA01030783 |
| 168.0786 | 168.0785 | 0.3 | 0.15 | C7H15NO(K)(H-1) | LMFA08020142 |
| 170.0154 | 170.0162 | -5 | 0.24 | C6H11OS(K)(H-1) | LMFA07060039 |
| 184.0737 | 184.0734 | 1.4 | 66.97 | C7H15NO2(K)(H-1) | LMFA01100013; LMFA01100015; LMFA01100014 |
| 190.0780 | 190.0787 | -3.7 | 0.69 | C10H17OS(Na)(H-2O-1)(H-1) | LMFA07060012 |
| 192.0935 | 192.0943 | -4.2 | 0.1 | C10H19OS(Na)(H-2O-1)(H-1) | LMFA07060013 |
| 198.0886 | 198.0891 | -2.2 | 3.28 | C8H17NO2(K)(H-1) | LMFA01100056 ; LMFA01100020 |
| 205.0829 | 205.0835 | -3 | 0.11 | C10H14O3(Na)(H-1) | LMPR0102070004 ; LMPR0102120042 |
| 205.0829 | 205.0835 | -3 | 0.11 | C10H14O3(Na)(H-1) | LMFA01030579; LMFA01060085 |
| 205.0829 | 205.0835 | -3 | 0.11 | C10H16O4(Na)(H-2O-1)(H-1) | LMFA01060079; LMFA01170132; LMFA01170131; LMFA01170032; LMFA01060080; LMFA01170045; LMFA01170044 |
| 205.0999 | 205.0989 | 5 | 0.22 | C11H20O2(K)(H-2O-1)(H-1) | LMPR0102010014 |
| 205.0999 | 205.0989 | 5 | 0.22 | C11H20O2(K)(H-2O-1)(H-1) | LMFA01030786; LMFA01030785; LMFA07010477; LMFA01030219; LMFA01030218; LMFA01030217; LMFA01030608; LMFA01030609; LMFA01020131; LMFA01020130; LMFA07010592; LMFA07040001; LMFA07010198; LMFA07010199;LMFA01030220; LMFA01030221; LMFA01030035 ; LMFA01030034 ; LMFA01030036 ; LMFA07010524; LMFA01030606; LMFA07040030 |
| 205.0999 | 205.0989 | 5 | 0.22 | C11H18O(K)(H-1) | LMFA06000068 |
| 207.0901 | 207.0911 | -4.9 | 0.15 | C11H19ClO(Na)(H-2O-1)(H-1) | LMFA05000143 |
| 207.1153 | 207.1144 | 4.1 | 0.11 | C14H16(Na)(H-1) | LMPR0103410002 |
| 207.1153 | 207.1146 | 3.3 | 0.11 | C11H22O2(K)(H-2O-1)(H-1) | LMFA07010454; LMFA07010437; LMFA01020155; LMFA07010571; LMFA07010606; LMFA01020090; LMFA07010200; LMFA07010595; LMFA07010196; LMFA01020182; LMFA01020183; LMFA01020184; LMFA01020185; LMFA01020186;LMFA01020187; LMFA01020188; LMFA07010408; LMFA07010653; LMFA01020346 ; LMFA07010521; LMFA07010529; LMFA01020261; LMFA12000158; LMFA01010011 ; LMFA01020288 |
| 207.1153 | 207.1146 | 3.3 | 0.11 | C11H20O(K)(H-1) | LMFA12000023; LMFA06000066; LMFA06000067; LMFA06000065; LMFA05000125; LMFA12000157 |
| 211.0012 | 211.0003 | 4 | 0.13 | C7H10O6(K)(H-2O-1)(H-1) | LMFA01170062; LMFA01170052 |
| 219.1237 | 219.1227 | 4.5 | 0.3 | C10H18O5 | LMFA01170092 ; LMFA01170076 |
| 220.1469 | 220.1462 | 3.1 | 0.28 | C12H25NO(K)(H-2O-1)(H-1) | LMFA08010001 |
| 223.0967 | 223.0965 | 1 | 0.12 | C12H16O5(H-2O-1) | LMFA01150004 |
| 223.0967 | 223.0965 | 1 | 0.12 | C12H14O4 | LMFA01170008 |
| 225.1050 | 225.1040 | 4.3 | 0.34 | C14H20O(K)(H-2O-1)(H-1) | LMFA05000016; LMFA06000180 |
| 225.1475 | 225.1485 | -4.3 | 0.14 | C13H20O3 | LMFA02020016 ; LMFA02020010 |
| 229.0590 | 229.0585 | 2.1 | 2.41 | C7H14N2O4(K)(H-1) | LMFA01170102 |
| 229.1225 | 229.1223 | 0.9 | 0.22 | C15H16O2 | LMPR0103300001 |
| 229.1225 | 229.1223 | 0.9 | 0.22 | C15H18O3(H-2O-1) | LMPR0103190001 ; LMPR0103190004 |
| 229.1590 | 229.1587 | 1.6 | 0.84 | C16H22O2(H-2O-1) | LMFA01140011 |
| 230.1441 | 230.1431 | 4.3 | 0.31 | C14H23(K)(H-1) | LMFA11000501 |
| 233.0273 | 233.0268 | 2.1 | 0.64 | C6H10O8(Na)(H-1) | LMFA01170108 ; LMFA01170107 |
| 234.1038 | 234.1049 | -4.7 | 0.15 | C12H21O2S(Na)(H-2O-1)(H-1) | LMFA07060014 |
| 234.1615 | 234.1619 | -1.6 | 0.19 | C13H27NO(K)(H-2O-1)(H-1) | LMFA08010012 |
| 237.1458 | 237.1461 | -1.5 | 0.12 | C12H22O3(Na)(H-1) | LMFA01060090; LMFA01060091; LMFA07040043; LMFA01050168; LMFA01050169; LMFA01060184; LMFA01060183; LMFA01060040 ; LMFA01060089; LMFA01060088; LMFA01050254; LMFA01050170; LMFA01060039 |
| 237.1471 | 237.1461 | 4.2 | 2.35 | C12H22O3(Na)(H-1) | LMFA01060090; LMFA01060091; LMFA07040043; LMFA01050168; LMFA01050169; LMFA01060184; LMFA01060183; LMFA01060040 ; LMFA01060089; LMFA01060088; LMFA01050254; LMFA01050170; LMFA01060039 |
| 239.1770 | 239.1770 | 0 | 0.54 | C16H24(Na)(H-1) | LMPR0103490003 B |
| 239.1770 | 239.1772 | -0.7 | 0.54 | C13H28O(K)(H-1) | LMFA05000171; LMFA05000523; LMFA05000035; LMFA05000514 |
| 239.1770 | 239.1770 | 0 | 0.54 | C16H26O(Na)(H-2O-1)(H-1) | LMFA06000196; LMFA06000198; LMFA06000199; LMFA06000213; LMFA06000226; LMFA06000096 |
| 243.1715 | 243.1719 | -1.7 | 0.36 | C15H24O(Na)(H-1) | LMPR0103830001 ; LMPR0103830003 ; LMPR0103660001 ; LMPR0103600002 ; LMPR0103010007 ; LMPR0103280002 ; LMPR0103560001 ; LMPR0103250001 ; LMPR0103370002 ; LMPR0103840001 ; LMPR0103700008 ; LMPR0103700009 ; LMPR0103700007 ; LMPR0103700004 ; LMPR0103010012 ; LMPR0103800003 ; LMPR0103800002 ; LMPR0103230001; LMPR0103270002 ; LMPR0103790001 ; LMPR0103780002 ; LMPR0103130001 ; LMPR0103140002 |
| 243.1715 | 243.1719 | -1.7 | 0.36 | C15H26O2(Na)(H-2O-1)(H-1) | LMPR0103530001 ; LMPR0103800001 ; LMPR0103710001 |
| 243.1715 | 243.1719 | -1.7 | 0.36 | C15H26O2(Na)(H-2O-1)(H-1) | LMFA07010554; LMFA07010269; LMFA01030790; LMFA07010538; LMFA07010631; LMFA07010615; LMFA07010617; LMFA07010526; LMFA07010277; LMFA07010270; LMFA07010279; LMFA07010278; LMFA01030581; LMFA01030582;LMFA07010625 |
| 243.1955 | 243.1955 | 0 | 0.38 | C14H28O4(H-2O-1) | LMFA01050081 |
| 243.1955 | 243.1955 | 0 | 0.38 | C14H26O3 | LMFA01060096; LMFA01060098; LMFA01060099; LMFA01060100; LMFA01050181; LMFA01050180; LMFA01060189; LMFA01060045 ; LMFA01060044 ; LMFA01060046 ;LMFA01050178; LMFA01050179 |
| 243.2082 | 243.2083 | -0.6 | 0.21 | C16H30O(Na)(H-2O-1)(H-1) | LMFA06000193; LMFA06000211; LMFA06000210; LMFA06000212; LMFA06000217; LMFA06000216; LMFA12000185; LMFA05000197; LMFA05000196; LMFA05000199; LMFA05000015; LMFA06000089; LMFA05000205; LMFA05000207;LMFA05000206; LMFA05000200; LMFA05000209; LMFA05000208; LMFA05000053; LMFA12000186; LMFA05000054; LMFA05000007 ; LMFA06000092; LMFA06000091; LMFA06000090; LMFA05000023; LMFA06000112 |
| 243.2082 | 243.2083 | -0.6 | 0.21 | C16H28(Na)(H-1) | LMFA11000047; LMFA11000368 |
| 245.1140 | 245.1148 | -3.4 | 0.29 | C13H18O3(Na)(H-1) | LMPR0103050009 |
| 245.1667 | 245.1666 | 0.3 | 0.19 | C15H28O(K)(H-2O-1)(H-1) | LMFA06000192; LMFA05000152; LMFA12000261; LMFA12000182; LMFA12000180; LMFA12000181; LMFA05000193; LMFA06000084; LMFA05000145; LMFA12000259; LMFA12000179 |
| 245.1667 | 245.1666 | 0.3 | 0.19 | C15H26(K)(H-1) | LMFA11000043; LMFA11000044; LMFA11000066; LMFA11000062; LMFA11000121; LMFA11000120; LMFA11000090; LMFA11000034 |
| 245.1905 | 245.1900 | 2 | 0.12 | C17H24O | LMFA05000019 ; LMFA05000530 |
| 245.1905 | 245.1900 | 2 | 0.12 | C17H26O2(H-2O-1) | LMFA01030475; LMFA01030477; LMFA01030476; LMFA01030478; LMFA01020368 |
| 249.1617 | 249.1615 | 0.5 | 1.08 | C14H28O2(K)(H-2O-1)(H-1) | LMFA01010053; LMFA07010661; LMFA01020191; LMFA01020190; LMFA07010439; LMFA01020159; LMFA01020158; LMFA01020272; LMFA07010262; LMFA01020070; LMFA01020035; LMFA01020036; LMFA07010448; LMFA07010464;LMFA01020189; LMFA07010422; LMFA01020160; LMFA01020161; LMFA01020388; LMFA01020069; LMFA01020007 ; LMFA01010014 ; LMFA01020285 |
| 249.1617 | 249.1614 | 1.1 | 1.08 | C17H24O(Na)(H-2O-1)(H-1) | LMFA05000019 ; LMFA05000530 |
| 249.1617 | 249.1615 | 0.5 | 1.08 | C14H26O(K)(H-1) | LMFA05000179; LMFA05000178; LMFA05000172; LMFA06000190; LMFA06000080; LMFA06000081; LMFA05000032; LMFA05000039; LMFA06000177; LMFA06000181; LMFA05000186; LMFA12000257; LMFA05000187; LMFA05000188;LMFA05000189; LMFA05000191; LMFA05000190; LMFA12000176; LMFA12000175; LMFA12000174; LMFA12000173; LMFA06000178; LMFA06000179; LMFA06000079; LMFA05000040; LMFA06000172 |
| 252.1916 | 252.1906 | 3.8 | 1.34 | C16H29OS(H-2O-1) | LMFA07060020 |
| 253.1776 | 253.1774 | 0.6 | 0.32 | C13H26O3(Na)(H-1) | LMFA01050326; LMFA01050042; LMFA01050040; LMFA01050041; LMFA01050172 |
| 253.2141 | 253.2138 | 1.3 | 1.63 | C14H30O2(Na)(H-1) | LMFA05000017 |
| 254.1299 | 254.1311 | -4.5 | 0.14 | C12H23O2S(Na)(H-1) | LMFA07060015 |
| 254.2071 | 254.2063 | 3.1 | 0.14 | C16H31OS(H-2O-1) | LMFA07060040 |
| 254.9789 | 254.9781 | 3 | 0.15 | C9H15BrO2(K)(H-2O-1)(H-1) | LMFA01090080; LMFA01090081 |
| 255.0794 | 255.0782 | 4.6 | 0.32 | C14H16O2(K)(H-1) | LMFA01030583 |
| 255.0794 | 255.0782 | 4.6 | 0.32 | C14H18O3(K)(H-2O-1)(H-1) | LMFA06000156 |
| 255.1963 | 255.1955 | 3.1 | 0.15 | C15H28O4(H-2O-1) | LMFA01170019; LMFA01170021 ; LMFA01170020 |
| 255.1963 | 255.1955 | 3.1 | 0.15 | C15H26O3 | LMFA07010489 |
| 255.2087 | 255.2083 | 1.4 | 0.92 | C17H28(Na)(H-1) | LMFA11000058 |
| 255.2087 | 255.2083 | 1.4 | 0.92 | C17H30O(Na)(H-2O-1)(H-1) | LMFA12000286; LMFA12000273; LMFA12000278; LMFA12000272; LMFA12000274; LMFA12000275; LMFA12000276; LMFA12000277; LMFA06000169 |
| 257.2283 | 257.2275 | 3.1 | 0.86 | C16H31FO2(H-2O-1) | LMFA01090034; LMFA01090041 |
| 258.1648 | 258.1648 | -0.1 | 0.23 | C14H25O2S | LMFA07060017 |
| 258.1693 | 258.1700 | -2.6 | 0.14 | C13H25NO5(H-2O-1) | LMFA00000002 |
| 260.2339 | 260.2349 | -3.9 | 0.22 | C16H33NO(Na)(H-2O-1)(H-1) | LMFA08010009 |
| 261.1814 | 261.1825 | -4.1 | 0.16 | C15H26O2(Na)(H-1) | LMPR0103530001 ; LMPR0103800001 ; LMPR0103710001 |
| 261.1814 | 261.1825 | -4.1 | 0.16 | C15H28O3(Na)(H-2O-1)(H-1) | LMFA01060102; LMFA01060103; LMFA01060049 ; LMFA01060048 ; LMFA01060047 |
| 261.1814 | 261.1825 | -4.1 | 0.16 | C15H26O2(Na)(H-1) | LMFA07010554; LMFA07010269; LMFA01030790; LMFA07010538; LMFA07010631; LMFA07010615; LMFA07010617; LMFA07010526; LMFA07010277; LMFA07010270; LMFA07010279; LMFA07010278; LMFA01030581; LMFA01030582;LMFA07010625 |
| 261.1860 | 261.1849 | 4.2 | 0.16 | C17H26O3(H-2O-1) | LMFA01050258; LMFA01060111; LMFA05000027 |
| 261.1860 | 261.1849 | 4.2 | 0.16 | C17H24O2 | LMFA05000529 ; LMFA05000005 ; LMFA05000028 ; LMFA05000022 |
| 261.1987 | 261.1979 | 2.9 | 4.35 | C16H32O(K)(H-2O-1)(H-1) | LMFA12000183; LMFA05000195; LMFA06000088 ; LMFA05000030; LMFA05000204; LMFA05000056; LMFA12000188; LMFA05000055; LMFA05000211; LMFA12000187; LMFA06000116 |
| 262.2533 | 262.2529 | 1.5 | 0.12 | C18H33NO(H-2O-1) | LMFA08010008 |
| 263.0889 | 263.0890 | -0.2 | 1.04 | C12H16O5(Na)(H-1) | LMFA01150004 |
| 263.2010 | 263.2006 | 1.6 | 0.38 | C17H26O2 | LMFA01030475; LMFA01030477; LMFA01030476; LMFA01030478; LMFA01020368 |
| 263.2010 | 263.2006 | 1.6 | 0.38 | C17H28O3(H-2O-1) | LMFA03050002 ; LMFA01070020 ; LMFA01050195 |
| 265.1558 | 265.1564 | -2.4 | 0.44 | C14H26O2(K)(H-1) | LMFA01030048 ; LMFA07010605; LMFA01030047; LMFA07010243; LMFA01030250; LMFA01020132; LMFA07010514; LMFA07010260; LMFA07010242; LMFA07010241; LMFA07010244; LMFA07010245; LMFA07010248;LMFA07010229; LMFA07010222; LMFA07010223; LMFA07010224; LMFA07010225; LMFA07010226; LMFA07010227; LMFA07040045 ; LMFA01030770 ; LMFA01030051 ;LMFA01030050; LMFA06000189; LMFA01030246; LMFA01030247; LMFA01030248 ; LMFA01030249; LMFA07010175; LMFA01020108; LMFA01030771 ; LMFA01030913; LMFA07040050;LMFA07040051 |
| 265.1558 | 265.1564 | -2.4 | 0.44 | C14H28O3(K)(H-2O-1)(H-1) | LMFA01050325; LMFA01050324; LMFA01050323; LMFA01050322; LMFA01050044 ; LMFA01050001 ; LMFA01050176; LMFA01050177; LMFA01050174; LMFA01050175; LMFA01050173 |
| 265.1558 | 265.1563 | -1.8 | 0.44 | C17H24O2(Na)(H-2O-1)(H-1) | LMFA05000529 ; LMFA05000005 ; LMFA05000028 ; LMFA05000022 |
| 266.2105 | 266.2115 | -3.7 | 0.32 | C16H27NO2 | LMSP01080050 |
| 266.2470 | 266.2478 | -3.1 | 0.4 | C17H33NO2(H-2O-1) | LMSP01080051 |
| 267.1344 | 267.1356 | -4.4 | 1.33 | C16H22O3(Na)(H-2O-1)(H-1) | LMPR0103520002 |
| 267.1344 | 267.1356 | -4.4 | 1.33 | C16H20O2(Na)(H-1) | LMFA01030706; LMFA01030703 |
| 267.1344 | 267.1357 | -5 | 1.33 | C13H24O3(K)(H-1) | LMFA01060097; LMFA07040044; LMFA01060041 ; LMFA01060043 ; LMFA01060042 |
| 268.1678 | 268.1673 | 1.8 | 0.81 | C13H27NO2(K)(H-1) | LMFA01100001; LMFA01100006; LMFA01100026; LMFA01100024 |
| 269.1346 | 269.1359 | -4.9 | 0.14 | C12H22O5(Na)(H-1) | LMFA01170089 |
| 270.2431 | 270.2428 | 1.2 | 0.44 | C16H33NO3(H-2O-1) | LMSP01080049 |
| 271.2401 | 271.2396 | 1.8 | 0.17 | C18H32(Na)(H-1) | LMFA11000167; LMFA11000381; LMFA11000174 |
| 271.2401 | 271.2396 | 1.8 | 0.17 | C18H34O(Na)(H-2O-1)(H-1) | LMFA12000285; LMFA06000232; LMFA06000231; LMFA06000230; LMFA06000237; LMFA06000236; LMFA06000238; LMFA05000057; LMFA06000100; LMFA05000047; LMFA12000199; LMFA06000099; LMFA05000217; LMFA05000214;LMFA05000048 |
| 273.2590 | 273.2577 | 4.9 | 0.14 | C20H32 | LMPR0104140001 ; LMPR0104160001 ; LMPR0104100001 ; LMPR0104240001 ; LMPR0104350003 ; LMPR0104080001 ; LMPR0104430001 ; LMPR0104280001 ; LMPR0104130002 ; LMPR0104080006 ; LMPR0104050007 ;LMPR0104050003 ; LMPR0104050002 ; LMPR0104410003 ; LMPR0104080005 ; LMPR0104150004 ; LMPR0104120001 ;LMPR0104030007 ; LMPR0104030008 ; LMPR0104080004 ; LMPR0104080007 ; LMPR0104480002 ; LMPR0104290001 ;LMPR0104190002 ; LMPR0104190001 ; LMPR0104390002 |
| 273.2590 | 273.2577 | 4.9 | 0.14 | C20H34O(H-2O-1) | LMPR0104530001 ; LMPR0104010018 ; LMPR0104160002 ; LMPR0103500014 ; LMPR0104010009 ; LMPR0104450002 ; LMPR0104380001 ; LMPR0104280002 ; LMPR0104220002 ; LMPR0104030005 |
| 273.2590 | 273.2577 | 4.9 | 0.14 | C20H34O(H-2O-1) | LMFA12000308; LMFA05000066 |
| 275.1975 | 275.1982 | -2.2 | 0.14 | C16H30O3(Na)(H-2O-1)(H-1) | LMFA01060058 ; LMFA01060056 ; LMFA01060057 ; LMFA01060054 ; LMFA01060055 ; LMFA01060052 ; LMFA01060053; LMFA01060050 ; LMFA01060051 ; LMFA01050106 ; LMFA01060104; LMFA01060105; LMFA01060101; LMFA01050271; LMFA01050270; LMFA01050272;LMFA01050192; LMFA01050193 |
| 275.1975 | 275.1982 | -2.2 | 0.14 | C16H28O2(Na)(H-1) | LMFA07010303; LMFA07010302; LMFA07010300; LMFA01030273; LMFA01030272; LMFA01030271; LMFA01030270; LMFA01030275 ; LMFA01030274; LMFA01030497; LMFA01030496; LMFA01030495; LMFA01030494;LMFA06000215; LMFA07010530; LMFA07010531; LMFA07010533; LMFA07010284; LMFA07010280; LMFA01030109; LMFA07010308; LMFA07010305; LMFA07010304; LMFA07010301; LMFA07010325; LMFA07010324; LMFA07010321;LMFA07010323; LMFA07010322; LMFA07010319; LMFA07010618; LMFA01030268; LMFA01030269; LMFA01030814; LMFA01030806 ; LMFA01030807 ; LMFA07010299; LMFA07010298;LMFA07010292; LMFA07010295; LMFA07010294; LMFA07010297; LMFA07010296; LMFA07010318; LMFA07010317; LMFA01140023 ; LMFA07010627 |
| 275.2213 | 275.2217 | -1.5 | 1.81 | C15H30O4 | LMGL01010008 |
| 275.2213 | 275.2217 | -1.5 | 1.81 | C15H30O4 | LMFA01050186; LMFA01050184; LMFA01050082 |
| 275.2339 | 275.2345 | -2.5 | 0.23 | C17H34O2(Na)(H-2O-1)(H-1) | LMPR0103010014 |
| 275.2339 | 275.2345 | -2.5 | 0.23 | C17H32O(Na)(H-1) | LMFA05000170; LMFA06000194; LMFA12000266; LMFA12000264; LMFA06000214; LMFA12000189; LMFA06000228; LMFA12000271; LMFA06000208; LMFA06000209; LMFA06000206; LMFA06000207; LMFA12000191; LMFA12000190;LMFA12000193; LMFA12000192 |
| 275.2339 | 275.2345 | -2.5 | 0.23 | C17H34O2(Na)(H-2O-1)(H-1) | LMFA07010602; LMFA07010470; LMFA01020193; LMFA01020197; LMFA01020196; LMFA01020195; LMFA01020194; LMFA01020199; LMFA01020198; LMFA07010415; LMFA01020390; LMFA01020092; LMFA07010326; LMFA07010342;LMFA01020012 ; LMFA01020011 ; LMFA07010596; LMFA07010677; LMFA07010425; LMFA07010488; LMFA07010487; LMFA01020265; LMFA01020202; LMFA01020201; LMFA01020200;LMFA01020041; LMFA01020040; LMFA01010017 ; LMFA01020281 |
| 276.2306 | 276.2298 | 3.1 | 1.37 | C16H33NO2(Na)(H-2O-1)(H-1) | LMSP01040008 |
| 276.2306 | 276.2298 | 3.1 | 1.37 | C16H33NO2(Na)(H-2O-1)(H-1) | LMFA01100017; LMFA01100016; LMFA01100018; LMFA08040042 |
| 276.2306 | 276.2298 | 3.1 | 1.37 | C16H31NO(Na)(H-1) | LMFA08010010 |
| 277.2160 | 277.2162 | -0.8 | 0.28 | C18H28O2 | LMST02010042 ; LMST02010050 |
| 277.2160 | 277.2162 | -0.8 | 0.28 | C18H28O2 | LMFA01030543; LMFA01030546; LMFA01030549; LMFA01030548; LMFA01030552; LMFA01030550; LMFA01030551; LMFA01030354; LMFA01030355 ; LMFA01030356; LMFA01030358; LMFA01030169; LMFA01030167;LMFA01030357 ; LMFA01030541; LMFA01030540; LMFA01030542; LMFA01030545; LMFA01030544; LMFA01030547; LMFA01030734; LMFA01030735; LMFA01030537; LMFA01030530; LMFA01030531; LMFA01030532;LMFA01030533; LMFA01030512; LMFA01030513; LMFA01030511; LMFA01030514; LMFA01030515; LMFA01030518; LMFA01030519; LMFA01030538; LMFA01030539; LMFA01030534; LMFA01030535; LMFA01030536; LMFA01030525;LMFA01030524; LMFA01030523; LMFA01030529; LMFA01030813; LMFA01030171 ; LMFA01030172; LMFA01030516; LMFA01030517; LMFA01140005; LMFA01030527; LMFA01030526; LMFA01030522; LMFA01030521;LMFA01030520; LMFA01030764 ; LMFA01030528 |
| 277.2160 | 277.2162 | -0.8 | 0.28 | C18H30O3(H-2O-1) | LMFA02000044 ; LMFA02000040 ; LMFA02000041 ; LMFA02000042 ; LMFA02000043 ; LMFA01150001; LMFA02000183 ;LMFA02000156 ; LMFA02000157 ; LMFA02000239 ; LMFA02000215; LMFA02000216; LMFA02000051 ; LMFA02000272; LMFA02000274 ;LMFA02000251 ; LMFA02000252 ; LMFA02000015 ; LMFA02010003 ; LMFA02010002 ; LMFA02010005 ; LMFA02010004 ; LMFA02010007 ; LMFA02010006 ; LMFA02000033 ; LMFA02000039 ; LMFA02000016 ; LMFA02000173 ; LMFA02000024 ; LMFA02000029 ; LMFA02000281 ; LMFA10000001 |
| 277.2524 | 277.2526 | -0.6 | 1.91 | C19H32O | LMST02020095 |
| 277.2524 | 277.2526 | -0.6 | 1.91 | C19H32O | LMPR0103500016 ;LMPR0103500006 ;LMPR0103500007 ;LMPR0103500009 |
| 277.2524 | 277.2526 | -0.6 | 1.91 | C19H34O2(H-2O-1) | LMFA01020379 ; LMFA01030129; LMFA01140018 ; LMFA01030899 ; LMFA01030365; LMFA01020378 ; LMFA01030503; LMFA07010621 |
| 278.2444 | 278.2454 | -3.6 | 0.32 | C16H35NO2(Na)(H-2O-1)(H-1) | LMSP01040001 |
| 278.2444 | 278.2454 | -3.6 | 0.32 | C16H33NO(Na)(H-1) | LMFA08010009 |
| 278.2490 | 278.2478 | 4 | 3.36 | C18H31NO | LMSP01080035 ; LMSP01080036 |
| 278.2490 | 278.2478 | 4 | 3.36 | C18H33NO2(H-2O-1) | LMSP01080013 |
| 279.1725 | 279.1719 | 2 | 0.15 | C18H26O2(Na)(H-2O-1)(H-1) | LMST02010044 |
| 279.1725 | 279.1719 | 2 | 0.15 | C18H26O2(Na)(H-2O-1)(H-1) | LMFA01030556; LMFA01030557; LMFA01030359; LMFA01030738; LMFA01030739; LMFA01030737; LMFA01030558; LMFA01030360; LMFA01140003; LMFA01030740 |
| 279.1725 | 279.1721 | 1.5 | 0.15 | C15H30O3(K)(H-2O-1)(H-1) | LMFA01050320; LMFA01050185; LMFA01050182; LMFA01050046 ; LMFA01050045; LMFA01050183; LMFA01050318; LMFA01050319 |
| 279.1725 | 279.1721 | 1.5 | 0.15 | C15H28O2(K)(H-1) | LMFA07010669; LMFA07010665; LMFA07010667; LMFA01030866 ; LMFA01030259; LMFA12000262; LMFA12000260; LMFA07010552; LMFA07010553; LMFA07010550; LMFA07010551; LMFA07010493; LMFA07010490;LMFA07010532; LMFA07010536; LMFA07010281; LMFA07010264; LMFA07010265; LMFA07010266; LMFA07010267; LMFA07010263; LMFA07010268; LMFA01030053; LMFA01030052; LMFA01030855 ; LMFA01020109;LMFA07010276; LMFA07010275; LMFA07010274; LMFA07010273; LMFA07010272; LMFA01030580 |
| 279.2456 | 279.2449 | 2.6 | 2.22 | C17H36(K)(H-1) | LMFA11000346; LMFA11000398; LMFA11000450; LMFA11000431; LMFA11000003 |
| 279.2664 | 279.2658 | 2 | 0.25 | C17H36O(Na)(H-1) | LMFA05000525; LMFA05000527; LMFA05000192; LMFA05000531; LMFA05000009 |
| 279.3032 | 279.3046 | -4.9 | 0.2 | C20H40O(H-2O-1) | LMPR0104010027 ; LMPR0104010002 |
| 279.3032 | 279.3046 | -4.9 | 0.2 | C20H40O(H-2O-1) | LMFA06000250; LMFA12000201; LMFA05000220; LMFA05000051; LMFA05000219 |
| 279.3032 | 279.3046 | -4.9 | 0.2 | C20H38 | LMFA11000128; LMFA11000139 |
| 280.2629 | 280.2635 | -1.9 | 0.13 | C18H35NO2(H-2O-1) | LMSP01080059; LMSP01080052 ; LMSP01080010  ; LMSP01080011  ; LMSP01080002 ; LMSP01010002 |
| 280.2629 | 280.2635 | -1.9 | 0.13 | C18H33NO | LMFA08010008 |
| 280.2629 | 280.2635 | -1.9 | 0.13 | C18H35NO2(H-2O-1) | LMFA08040043 |
| 281.2075 | 281.2087 | -4.3 | 0.43 | C15H30O3(Na)(H-1) | LMFA01050320; LMFA01050185; LMFA01050182; LMFA01050046 ; LMFA01050045; LMFA01050183; LMFA01050318; LMFA01050319 |
| 283.1890 | 283.1880 | 3.7 | 1.04 | C14H28O4(Na)(H-1) | LMFA01050081 |
| 283.1890 | 283.1904 | -4.7 | 1.04 | C16H28O5(H-2O-1) | LMFA03010213 |
| 285.2056 | 285.2060 | -1.6 | 0.25 | C16H30O5(H-2O-1) | LMFA01170058; LMFA01170057 |
| 287.2359 | 287.2369 | -3.7 | 0.48 | C20H32O2(H-2O-1) | LMST02020107 ; LMST02020012 ; LMST02020027 |
| 287.2359 | 287.2345 | 4.7 | 0.48 | C18H32O(Na)(H-1) | LMPR0103500010 |
| 287.2359 | 287.2369 | -3.7 | 0.48 | C20H32O2(H-2O-1) | LMPR0104210003 ; LMPR0104530002 ; LMPR0104250002 ; LMPR0104150006 ; LMPR0104470001 ; LMPR0104250001 ; LMPR0104400003 ;LMPR0104390005 |
| 287.2359 | 287.2369 | -3.7 | 0.48 | C20H30O | LMPR01090009 ; LMPR0104140002 ; LMPR01090001 ; LMPR0104080009 ; LMPR0104050010 ; LMPR01090005 ; LMPR0104130005 ; LMPR0104070001 ; LMPR0104050009 ; LMPR0104110003 ; LMPR01090011 ; LMPR0104020001 |
| 287.2359 | 287.2369 | -3.7 | 0.48 | C20H30O | LMST02030210 |
| 287.2359 | 287.2345 | 4.7 | 0.48 | C18H34O2(Na)(H-2O-1)(H-1) | LMFA01030068; LMFA01030069; LMFA01030062 ; LMFA01030063 ; LMFA01030061; LMFA01030066 ; LMFA01030067 ; LMFA01030064 ;LMFA01030065 ; LMFA01030002 ; LMFA01020373 ; LMFA01020372 ; LMFA01020374 ; LMFA07010498; LMFA07010497; LMFA01030080; LMFA01030081;LMFA07010343; LMFA07010346; LMFA07010347; LMFA07010345; LMFA07010344; LMFA01030895 ; LMFA07010633; LMFA07010378; LMFA01030075; LMFA01030074 ; LMFA01030077 ;LMFA01030076 ; LMFA01030071 ; LMFA01030070 ; LMFA01030073 ; LMFA01030079 ; LMFA01030078 ; LMFA01030295; LMFA01030294;LMFA01030290; LMFA01030293; LMFA01030292; LMFA07010368; LMFA07010361; LMFA07010363; LMFA07010362; LMFA07010365; LMFA07010367; LMFA07010366; LMFA07010500; LMFA07010502; LMFA01020206; LMFA01140008;LMFA07040056; LMFA01030296; LMFA01030291; LMFA01030880 ; LMFA01030881 ; LMFA01030882 |
| 287.2359 | 287.2369 | -3.7 | 0.48 | C20H32O2(H-2O-1) | LMFA01030688; LMFA01030689; LMFA01030687; LMFA01030001 ; LMFA01030390; LMFA01030391; LMFA01030392; LMFA01030393; LMFA01030394 ; LMFA01030395; LMFA01030905 ; LMFA01030906 ; LMFA01030871 ; LMFA01020367 ; LMFA01030818 ; LMFA01030817; LMFA01030816; LMFA01030389; LMFA01030175; LMFA01030176; LMFA01030173; LMFA01020381 ; LMFA01140006; LMFA01020222 |
| 287.2359 | 287.2345 | 4.7 | 0.48 | C18H32O(Na)(H-1) | LMFA12000284; LMFA12000287; LMFA12000288; LMFA06000233; LMFA06000235; LMFA06000234; LMFA06000239; LMFA06000101; LMFA06000242; LMFA06000240; LMFA06000241; LMFA05000216 |
| 288.2528 | 288.2533 | -1.6 | 1.11 | C16H33NO3 | LMSP01080049 |
| 289.1567 | 289.1564 | 1.1 | 0.23 | C16H28O3(K)(H-2O-1)(H-1) | LMFA02010014; LMFA02010015 |
| 289.1567 | 289.1564 | 1.1 | 0.23 | C16H26O2(K)(H-1) | LMFA07010306; LMFA01030869 ; LMFA07010320; LMFA01030134; LMFA01030135; LMFA01030136; LMFA01030137 ; LMFA01030138 ; LMFA01030139; LMFA01030702;LMFA07010584 |
| 289.2489 | 289.2502 | -4.3 | 0.63 | C18H36O2(Na)(H-2O-1)(H-1) | LMFA07010457; LMFA07010450; LMFA07010601; LMFA07010471; LMFA07010473; LMFA01020175; LMFA01020094 ; LMFA01020093 ; LMFA01020014 ; LMFA01020013; LMFA07010597;LMFA07010441; LMFA07010466; LMFA07010379; LMFA07010679; LMFA01020292 ; LMFA07010426; LMFA07010527; LMFA01020205; LMFA01020045; LMFA01020044; LMFA01020046; LMFA01020043;LMFA01020042; LMFA01010018 |
| 289.2489 | 289.2502 | -4.3 | 0.63 | C18H34O(Na)(H-1) | LMFA12000285; LMFA06000232; LMFA06000231; LMFA06000230; LMFA06000237; LMFA06000236; LMFA06000238; LMFA05000057; LMFA06000100; LMFA05000047; LMFA12000199; LMFA06000099; LMFA05000217; LMFA05000214;LMFA05000048 |
| 289.2534 | 289.2526 | 2.8 | 1.52 | C20H34O2(H-2O-1) | LMPR0104010010 |
| 289.2534 | 289.2526 | 2.8 | 1.52 | C20H32O | LMPR0104080008 ; LMPR01090047 ; LMPR0104420003 ; LMPR0104460001 ; LMPR0104450003 ; LMPR0104130003 ; LMPR0104050006 ; LMPR0104050008 ; LMPR0104400002 ; LMPR0104390003 |
| 289.2534 | 289.2526 | 2.8 | 1.52 | C20H34O2(H-2O-1) | LMFA01030863 ; LMFA01030378 ; LMFA01030379; LMFA01030158 ; LMFA01030159; LMFA01030383; LMFA01030382; LMFA01030381 ; LMFA01030380; LMFA01030386; LMFA01030385; LMFA01030384; LMFA01030388; LMFA07010544; LMFA07010542; LMFA01030157; LMFA01020221; LMFA01140021 ; LMFA07010395; LMFA07010396; LMFA01030765; LMFA01030767 |
| 289.2534 | 289.2526 | 2.8 | 1.52 | C20H32O | LMFA06000197; LMFA05000064 |
| 290.2450 | 290.2454 | -1.3 | 0.19 | C17H35NO2(Na)(H-2O-1)(H-1) | LMSP01040002 ; LMSP01080005  ; LMSP01080006 |
| 290.2450 | 290.2454 | -1.3 | 0.19 | C17H35NO2(Na)(H-2O-1)(H-1) | LMFA08040045 |
| 291.2456 | 291.2449 | 2.6 | 0.38 | C18H38O(K)(H-2O-1)(H-1) | LMFA05000085 ; LMFA05000044 |
| 291.2456 | 291.2449 | 2.6 | 0.38 | C18H36(K)(H-1) | LMFA11000325; LMFA11000096; LMFA11000563; LMFA11000113 |
| 291.2663 | 291.2658 | 1.6 | 0.33 | C18H38O2(Na)(H-2O-1)(H-1) | LMFA05000072 ; LMFA05000070 ; LMFA05000071 |
| 291.2663 | 291.2658 | 1.6 | 0.33 | C18H36O(Na)(H-1) | LMFA12000280; LMFA12000289; LMFA05000095 ; LMFA12000279; LMFA06000098 ; LMFA05000212; LMFA05000213; LMFA05000215 |
| 292.2621 | 292.2635 | -4.6 | 0.19 | C19H35NO2(H-2O-1) | LMSP01080014 |
| 292.2621 | 292.2611 | 3.6 | 0.19 | C17H37NO2(Na)(H-2O-1)(H-1) | LMSP01040003 |
| 293.2833 | 293.2839 | -2 | 0.27 | C20H38O2(H-2O-1) | LMPR0104010024 ; LMPR0104010023 ; LMPR0104010005 |
| 293.2833 | 293.2839 | -2 | 0.27 | C20H38O2(H-2O-1) | LMFA07010609; LMFA01030372; LMFA01030370; LMFA01030371; LMFA01030082; LMFA01030084 ; LMFA01030085 ; LMFA01030086 ; LMFA01030087; LMFA03010000 ; LMFA07010387; LMFA07010383; LMFA07010382; LMFA07010389; LMFA07010388; LMFA01030858 ; LMFA01030369; LMFA01030368; LMFA01030367; LMFA01030366; LMFA01030700; LMFA07040058; LMFA07010397 |
| 293.2833 | 293.2839 | -2 | 0.27 | C20H36O | LMFA12000313; LMFA12000312; LMFA12000311; LMFA12000310; LMFA12000309; LMFA05000210 |
| 294.2790 | 294.2791 | -0.6 | 0.24 | C19H37NO2(H-2O-1) | LMSP01080012 |
| 296.2960 | 296.2948 | 4 | 0.13 | C19H39NO2(H-2O-1) | LMFA08040049 |
| 299.0678 | 299.0680 | -0.6 | 0.28 | C15H16O4(K)(H-1) | LMPR0103330004 |
| 300.1561 | 300.1571 | -3.2 | 1.46 | C11H26NO6P | LMGP01040067 |
| 301.2370 | 301.2373 | -1.3 | 0.17 | C17H32O4 | LMFA01170028 ; LMFA01170025; LMFA01170024; LMFA01170027; LMFA01170026; LMFA01170023 |
| 301.2735 | 301.2737 | -0.8 | 1.12 | C18H36O3 | LMFA02000185; LMFA02000129 ; LMFA02000123 ; LMFA02000122 ; LMFA02000121 ; LMFA02000120 ;LMFA02000127 ; LMFA02000126 ; LMFA02000125 ; LMFA02000124 ; LMFA02000237 ; LMFA02000233;LMFA02000232; LMFA02000198; LMFA02000196; LMFA02000197; LMFA02000195 ; LMFA02000128 ; LMFA02000130 ; LMFA02000131 ;LMFA02000132 ; LMFA02000133 ; LMFA02000134 ; LMFA02000135 ; LMFA02000136 |
| 303.1957 | 303.1955 | 0.7 | 0.34 | C19H26O3 | LMPR0102060006 ; LMPR0102060007 ; LMPR0102060008 ; LMPR0102060009 ; LMPR0102060017 ; LMPR0102060016 ;LMPR0102060015 ; LMPR0102060014 ; LMPR0102060013 ; LMPR0102060012 ; LMPR0102060011 ; LMPR0102060010 |
| 303.1957 | 303.1955 | 0.7 | 0.34 | C19H28O4(H-2O-1) | LMST02020081 |
| 303.1957 | 303.1955 | 0.7 | 0.34 | C19H26O3 | LMST02010035 ; LMST02020061; LMST02020066; LMST02020067; LMST02020065; LMST02020071; LMST02010051 |
| 303.2644 | 303.2658 | -4.9 | 1.29 | C19H38O2(Na)(H-2O-1)(H-1) | LMPR0104010022 |
| 303.2644 | 303.2658 | -4.9 | 1.29 | C19H38O2(Na)(H-2O-1)(H-1) | LMFA07010474; LMFA01020177; LMFA01020176; LMFA01020096; LMFA01020095; LMFA01020210; LMFA01020211; LMFA01020212; LMFA01020213; LMFA01020214; LMFA01020215; LMFA01020216; LMFA01020217; LMFA01020218;LMFA01020219; LMFA01020016 ; LMFA01020015 ; LMFA07010381; LMFA07010675; LMFA07010427; LMFA01020047; LMFA07010582; LMFA01010019 |
| 303.2644 | 303.2658 | -4.9 | 1.29 | C19H36O(Na)(H-1) | LMFA12000281; LMFA12000204; LMFA12000207; LMFA12000203; LMFA12000269; LMFA12000292; LMFA12000293; LMFA12000291; LMFA06000246; LMFA06000245 |
| 304.2598 | 304.2611 | -4.3 | 0.64 | C18H37NO2(Na)(H-2O-1)(H-1) | LMSP01020002 ; LMSP01080004 ; LMSP01080008  ; LMSP01010001 |
| 304.2598 | 304.2611 | -4.3 | 0.64 | C18H35NO(Na)(H-1) | LMFA08010004 ; LMFA08010011 |
| 304.2598 | 304.2611 | -4.3 | 0.64 | C18H37NO2(Na)(H-2O-1)(H-1) | LMFA08040013 ; LMFA01100008; LMFA01100009 |
| 305.2116 | 305.2111 | 1.6 | 1.67 | C19H28O3 | LMST02020088 ; LMST02020087 ; LMST02020092 ; LMST02020091 ; LMST02020090 ; LMST02020054; LMST02020068 ; LMST02020064 ; LMST02020102 ; LMST02020106 ; LMST02020070;LMST02020109; LMST02020101 |
| 305.2327 | 305.2323 | 1.5 | 0.16 | C16H32O5 | LMFA01050099 ; LMFA01050102 ; LMFA01050101 ; LMFA01050100 |
| 305.2804 | 305.2815 | -3.5 | 1.41 | C19H38O(Na)(H-1) | LMPR0104010012 |
| 305.2804 | 305.2815 | -3.5 | 1.41 | C19H40O2(Na)(H-2O-1)(H-1) | LMFA05000073 ; LMFA05000074 |
| 305.2804 | 305.2815 | -3.5 | 1.41 | C19H38O(Na)(H-1) | LMFA12000283; LMFA12000282; LMFA12000200; LMFA12000202; LMFA12000268; LMFA06000247; LMFA12000051; LMFA12000198; LMFA12000197; LMFA12000304; LMFA12000303 |
| 306.2757 | 306.2767 | -3.3 | 0.71 | C18H39NO2(Na)(H-2O-1)(H-1) | LMSP01080058 ; LMSP01080055 ; LMSP01020001 |
| 306.2757 | 306.2767 | -3.3 | 0.71 | C18H37NO(Na)(H-1) | LMFA08010003 |
| 307.2273 | 307.2268 | 1.8 | 1.93 | C19H30O3 | LMST02020086 ; LMST02020099 ; LMST02020098; LMST02020097; LMST02020004 |
| 307.2273 | 307.2268 | 1.8 | 1.93 | C19H32O4(H-2O-1) | LMFA01040038; LMFA01040039 |
| 308.2225 | 308.2220 | 1.4 | 1.09 | C18H31NO4(H-2O-1) | LMFA01120002; LMFA01120001; LMFA08030007 |
| 308.2225 | 308.2220 | 1.4 | 1.09 | C18H29NO3 | LMFA08020085 |
| 308.2914 | 308.2924 | -3.1 | 0.6 | C18H39NO(Na)(H-1) | LMSP01080032 |
| 309.2179 | 309.2190 | -3.8 | 0.19 | C17H34O2(K)(H-1) | LMPR0103010014 |
| 309.2179 | 309.2189 | -3.3 | 0.19 | C20H32O2(Na)(H-2O-1)(H-1) | LMPR0104210003 ; LMPR0104530002 ; LMPR0104250002 ; LMPR0104150006 ; LMPR0104470001 ; LMPR0104250001 ; LMPR0104400003 ;LMPR0104390005 |
| 309.2179 | 309.2189 | -3.3 | 0.19 | C20H30O(Na)(H-1) | LMPR01090009 ; LMPR0104140002 ; LMPR01090001 ; LMPR0104080009 ; LMPR0104050010 ; LMPR01090005 ; LMPR0104130005 ; LMPR0104070001 ; LMPR0104050009 ; LMPR0104110003 ; LMPR01090011 ; LMPR0104020001 |
| 309.2179 | 309.2189 | -3.3 | 0.19 | C20H32O2(Na)(H-2O-1)(H-1) | LMST02020107 ; LMST02020012 ; LMST02020027 |
| 309.2179 | 309.2189 | -3.3 | 0.19 | C20H30O(Na)(H-1) | LMST02030210 |
| 309.2179 | 309.2189 | -3.3 | 0.19 | C20H32O2(Na)(H-2O-1)(H-1) | LMFA01030688; LMFA01030689; LMFA01030687; LMFA01030001 ; LMFA01030390; LMFA01030391; LMFA01030392; LMFA01030393; LMFA01030394 ; LMFA01030395; LMFA01030905 ; LMFA01030906 ; LMFA01030871 ; LMFA01020367 ; LMFA01030818 ; LMFA01030817; LMFA01030816; LMFA01030389; LMFA01030175; LMFA01030176; LMFA01030173; LMFA01020381 ; LMFA01140006; LMFA01020222 |
| 309.2179 | 309.2190 | -3.8 | 0.19 | C17H34O2(K)(H-1) | LMFA07010602; LMFA07010470; LMFA01020193; LMFA01020197; LMFA01020196; LMFA01020195; LMFA01020194; LMFA01020199; LMFA01020198; LMFA07010415; LMFA01020390; LMFA01020092; LMFA07010326; LMFA07010342;LMFA01020012 ; LMFA01020011 ; LMFA07010596; LMFA07010677; LMFA07010425; LMFA07010488; LMFA07010487; LMFA01020265; LMFA01020202; LMFA01020201; LMFA01020200;LMFA01020041; LMFA01020040; LMFA01010017 ; LMFA01020281 |
| 309.2429 | 309.2424 | 1.5 | 2.39 | C19H32O3 | LMFA01070014; LMFA01070013; LMFA01070012 |
| 309.2429 | 309.2424 | 1.5 | 2.39 | C19H34O4(H-2O-1) | LMFA01080002; LMFA01080007; LMFA01170126 |
| 309.2753 | 309.2764 | -3.6 | 0.27 | C18H38O2(Na)(H-1) | LMFA05000072 ; LMFA05000070 ; LMFA05000071 |
| 310.2143 | 310.2143 | -0.1 | 0.13 | C16H33NO2(K)(H-1) | LMSP01040008 |
| 310.2143 | 310.2143 | -0.1 | 0.13 | C16H33NO2(K)(H-1) | LMFA01100017; LMFA01100016; LMFA01100018; LMFA08040042 |
| 310.2380 | 310.2377 | 1.2 | 1.99 | C18H33NO4(H-2O-1) | LMFA01120003; LMFA01120004 |
| 310.2703 | 310.2717 | -4.3 | 0.21 | C17H37NO2(Na)(H-1) | LMSP01040003 |
| 311.2333 | 311.2345 | -4 | 0.59 | C20H32O(Na)(H-1) | LMPR0104080008 ; LMPR01090047 ; LMPR0104420003 ; LMPR0104460001 ; LMPR0104450003 ; LMPR0104130003 ; LMPR0104050006 ; LMPR0104050008 ; LMPR0104400002 ; LMPR0104390003 |
| 311.2333 | 311.2345 | -4 | 0.59 | C20H34O2(Na)(H-2O-1)(H-1) | LMPR0104010010 |
| 311.2333 | 311.2345 | -4 | 0.59 | C20H34O2(Na)(H-2O-1)(H-1) | LMFA01030863 ; LMFA01030378 ; LMFA01030379; LMFA01030158 ; LMFA01030159; LMFA01030383; LMFA01030382; LMFA01030381 ; LMFA01030380; LMFA01030386; LMFA01030385; LMFA01030384; LMFA01030388; LMFA07010544; LMFA07010542; LMFA01030157; LMFA01020221; LMFA01140021 ; LMFA07010395; LMFA07010396; LMFA01030765; LMFA01030767 |
| 311.2333 | 311.2347 | -4.5 | 0.59 | C17H36O2(K)(H-1) | LMFA05000069 |
| 311.2333 | 311.2345 | -4 | 0.59 | C20H32O(Na)(H-1) | LMFA06000197; LMFA05000064 |
| 312.2293 | 312.2298 | -1.6 | 0.14 | C19H33NO2(Na)(H-2O-1)(H-1) | LMSP01080043 |
| 312.2293 | 312.2299 | -2.1 | 0.14 | C16H35NO2(K)(H-1) | LMSP01040001 |
| 313.2736 | 313.2737 | -0.5 | 2.99 | C19H38O4(H-2O-1) | LMGL01010009 ; LMGL01010001 ; LMGL01010025 |
| 313.2736 | 313.2737 | -0.5 | 2.99 | C19H36O3 | LMFA01060128; LMFA01060129; LMFA01060131; LMFA01060130 |
| 314.2441 | 314.2454 | -4.3 | 0.13 | C19H35NO2(Na)(H-2O-1)(H-1) | LMSP01080014 |
| 314.2685 | 314.2690 | -1.4 | 1.95 | C18H35NO3 | LMFA08020079 |
| 315.2888 | 315.2894 | -1.9 | 1.16 | C19H38O3 | LMFA01050072; LMFA01050071 |
| 316.2024 | 316.2037 | -4.2 | 1.14 | C18H33NO2(K)(H-2O-1)(H-1) | LMSP01080013 |
| 316.2024 | 316.2037 | -4.2 | 1.14 | C18H31NO(K)(H-1) | LMSP01080035 ; LMSP01080036 |
| 316.2837 | 316.2846 | -3 | 0.99 | C18H37NO3 | LMSP01030002 ; LMSP01080003 ; LMSP01080009 |
| 317.0797 | 317.0786 | 3.5 | 0.16 | C15H20O6(K)(H-2O-1)(H-1) | LMPR0103180002 |
| 317.1974 | 317.1959 | 4.9 | 0.15 | C16H30O7(H-2O-1) | LMFA13030003 |
| 319.2120 | 319.2115 | 1.5 | 0.53 | C16H30O6 | LMPR0102090060 |
| 320.2359 | 320.2350 | 2.8 | 0.29 | C18H37NO2(K)(H-2O-1)(H-1) | LMSP01020002 ; LMSP01080004 ; LMSP01080008  ; LMSP01010001 |
| 320.2359 | 320.2350 | 2.8 | 0.29 | C18H35NO(K)(H-1) | LMFA08010004 ; LMFA08010011 |
| 320.2359 | 320.2350 | 2.8 | 0.29 | C18H37NO2(K)(H-2O-1)(H-1) | LMFA08040013 ; LMFA01100008; LMFA01100009 |
| 323.1756 | 323.1772 | -4.7 | 0.48 | C20H30O2(K)(H-2O-1)(H-1) | LMST02020044 ; LMST02020028 ; LMST02020029 |
| 323.1756 | 323.1772 | -4.9 | 0.48 | C19H27ClO2 | LMST02020019 |
| 323.1756 | 323.1772 | -4.7 | 0.48 | C20H30O2(K)(H-2O-1)(H-1) | LMPR0104210001 ; LMPR0104100002 ; LMPR0104080010 ; LMPR0104500001 ; LMPR0104130004 ; LMPR0104050004 ;LMPR0104050001 ; LMPR0104110001 ; LMPR0104200001 ; LMPR0104400004 |
| 323.1756 | 323.1772 | -4.7 | 0.48 | C20H28O(K)(H-1) | LMPR01090003 ; LMPR01090035 ; LMPR01090036 ; LMPR0104050011 ; LMPR01090008 ; LMPR01090002 ;LMPR0104410002 ; LMPR01090018 ; LMPR01090017 ; LMPR01090010 |
| 323.1756 | 323.1772 | -4.7 | 0.48 | C20H28O(K)(H-1) | LMST02030127 |
| 323.1756 | 323.1772 | -4.7 | 0.48 | C20H30O2(K)(H-2O-1)(H-1) | LMFA01030396; LMFA01030397; LMFA01030759 ; LMFA01030180; LMFA01030693; LMFA01030695; LMFA01030694; LMFA01140004; LMFA01030760 |
| 324.1520 | 324.1520 | -0.1 | 0.67 | C16H29O2S(K)(H-1) | LMFA07060019 |
| 327.1490 | 327.1486 | 1 | 0.28 | C19H27ClO2(Na)(H-2O-1)(H-1) | LMST02020019 |
| 327.1490 | 327.1488 | 0.6 | 0.28 | C16H31ClO3(K)(H-2O-1)(H-1) | LMFA01090054; LMFA01090044 |
| 327.2652 | 327.2658 | -1.8 | 0.3 | C21H36O(Na)(H-1) | LMST02030174 |
| 327.2652 | 327.2658 | -1.8 | 0.3 | C21H38O2(Na)(H-2O-1)(H-1) | LMFA01030862 ; LMFA01030868 ; LMFA01030399; LMFA12000314; LMFA12000219; LMFA12000317; LMFA12000316; LMFA12000318; LMFA01020225; LMFA01020224; LMFA01020226 |
| 327.2652 | 327.2658 | -1.8 | 0.3 | C21H36O(Na)(H-1) | LMFA12000319 |
| 327.2696 | 327.2682 | 4 | 0.74 | C23H36O2(H-2O-1) | LMST03020018 ; LMST03020019 ;LMST03020016 ; LMST03020017 ; LMST03020014 ; LMST03020015 ; LMST01010302; LMST03020650 |
| 327.2696 | 327.2682 | 4 | 0.74 | C23H36O2(H-2O-1) | LMPR02010037 |
| 328.2596 | 328.2611 | -4.6 | 0.28 | C20H35NO(Na)(H-1) | LMSP01080053 |
| 328.2596 | 328.2611 | -4.6 | 0.28 | C20H37NO2(Na)(H-2O-1)(H-1) | LMFA08040004 |
| 328.2639 | 328.2635 | 1.3 | 0.44 | C22H35NO2(H-2O-1) | LMFA08040008 |
| 328.2848 | 328.2846 | 0.7 | 0.22 | C19H37NO3 | LMFA08020123 |
| 329.2836 | 329.2839 | -0.9 | 0.61 | C23H38O2(H-2O-1) | LMST04060014; LMST04060016; LMST04060013; LMST04060015 |
| 329.2836 | 329.2850 | -4.3 | 0.61 | C20H37FO2 | LMFA01090053; LMFA01090052 |
| 330.2047 | 330.2040 | 2.3 | 0.27 | C18H31NO4(Na)(H-2O-1)(H-1) | LMFA01120002; LMFA01120001; LMFA08030007 |
| 330.2047 | 330.2040 | 2.3 | 0.27 | C18H29NO3(Na)(H-1) | LMFA08020085 |
| 330.2778 | 330.2791 | -4.2 | 0.49 | C22H37NO2(H-2O-1) | LMFA00000014 ; LMFA08040001 |
| 330.2778 | 330.2767 | 3.1 | 0.49 | C20H39NO2(Na)(H-2O-1)(H-1) | LMFA08040015 |
| 331.2596 | 331.2608 | -3.6 | 0.15 | C20H36O2(Na)(H-1) | LMPR0104030010 |
| 331.2596 | 331.2608 | -3.6 | 0.15 | C20H38O3(Na)(H-2O-1)(H-1) | LMPR0104010016 |
| 331.2596 | 331.2608 | -3.6 | 0.15 | C20H36O2(Na)(H-1) | LMFA01030860 ; LMFA01030867 ; LMFA01030865 ; LMFA01030373; LMFA01030376; LMFA01030377; LMFA01030374; LMFA01030375; LMFA01030900 ; LMFA07010386; LMFA07010385;LMFA07010384; LMFA01030898 ; LMFA07010612; LMFA01030859 ; LMFA01030130; LMFA07010394; LMFA07010390; LMFA07010391; LMFA07010392; LMFA07010393; LMFA07010624 |
| 331.2596 | 331.2608 | -3.6 | 0.15 | C20H38O3(Na)(H-2O-1)(H-1) | LMFA01050257; LMFA01050256; LMFA01050117 ; LMFA01060134; LMFA01060133; LMFA01060132 |
| 331.2847 | 331.2843 | 1.4 | 0.48 | C19H38O4 | LMGL01010009 ; LMGL01010001 ; LMGL01010025 |
| 331.2973 | 331.2971 | 0.5 | 0.23 | C21H42O2(Na)(H-2O-1)(H-1) | LMPR02020064 |
| 331.2973 | 331.2971 | 0.5 | 0.23 | C21H42O2(Na)(H-2O-1)(H-1) | LMFA07010453; LMFA07010416; LMFA07010576; LMFA01020356 ; LMFA07010556; LMFA01020033; LMFA01020018; LMFA01010021; LMFA07010443; LMFA07010649; LMFA07010509; LMFA01020223 |
| 331.2973 | 331.2971 | 0.5 | 0.23 | C21H40O(Na)(H-1) | LMFA12000315; LMFA12000211; LMFA12000214; LMFA12000215; LMFA05000533; LMFA05000534 |
| 332.2186 | 332.2196 | -3.2 | 0.46 | C18H33NO4(Na)(H-2O-1)(H-1) | LMFA01120003; LMFA01120004 |
| 332.2915 | 332.2924 | -2.7 | 0.21 | C20H41NO2(Na)(H-2O-1)(H-1) | LMSP01070001 |
| 332.2915 | 332.2924 | -2.7 | 0.21 | C20H41NO2(Na)(H-2O-1)(H-1) | LMFA08040051 |
| 333.0628 | 333.0615 | 3.8 | 0.19 | C15H22BrClO | LMPR0103670002 |
| 333.2044 | 333.2060 | -4.9 | 0.16 | C20H30O5(H-2O-1) | LMPR0104070002 |
| 333.2044 | 333.2060 | -4.9 | 0.16 | C20H28O4 | LMPR0104170014 ; LMPR0104170010 ; LMPR0104170029 ; LMPR0104420002 |
| 333.2044 | 333.2036 | 2.3 | 0.16 | C18H30O4(Na)(H-1) | LMFA02000247; LMFA02000279; LMFA02000109 ; LMFA02000108 ; LMFA02000052 ; LMFA02000019; LMFA02000018 ; LMFA02000030 ; LMFA02000226 ;LMFA02000020 ; LMFA02000114 ; LMFA02000112 ; LMFA02000110 ; LMFA02000111 |
| 333.2044 | 333.2060 | -4.9 | 0.16 | C20H30O5(H-2O-1) | LMFA03010130 ; LMFA03010132 ; LMFA03010135 ; LMFA03060099 ; LMFA03030005 ; LMFA03010045 ; LMFA03040009 ; LMFA03070042; LMFA03070041 ; LMFA03010023 ; LMFA03110009 ; LMFA03010167 ; LMFA03010166 ; LMFA03010142 ; LMFA01030748;LMFA03010206 ; LMFA03010205 ; LMFA03070039 ; LMFA03070035 ; LMFA03070034 ; LMFA03010030 ; LMFA03070019 |
| 333.2044 | 333.2036 | 2.3 | 0.16 | C18H32O5(Na)(H-2O-1)(H-1) | LMFA03010154; LMFA02000105; LMFA02000106; LMFA02000021; LMFA02000022; LMFA02000220 |
| 333.2044 | 333.2060 | -4.9 | 0.16 | C20H28O4 | LMFA03010199 ; LMFA03070040 ; LMFA03070026 ; LMFA03010141 ; LMFA03010140 ; LMFA03010180 |
| 333.2621 | 333.2636 | -4.4 | 0.2 | C18H36O5 | LMFA02000005 ; LMFA02000006 ; LMFA02000147 |
| 335.2181 | 335.2193 | -3.5 | 0.42 | C18H32O4(Na)(H-1) | LMFA02000045 ; LMFA02000046 ; LMFA02000047 ; LMFA02000048 ; LMFA02000049 ; LMFA02000264; LMFA02000064 ; LMFA02000248; LMFA02000009; LMFA02000167 ; LMFA02000166 ; LMFA02000101 ; LMFA02000100 ; LMFA02000103 ; LMFA02000102 ; LMFA02000104; LMFA02000249; LMFA01170055; LMFA02000250; LMFA02000099 ; LMFA02000050 ; LMFA02000277; LMFA02000278; LMFA02000013; LMFA02000012 ; LMFA02000034 ; LMFA02000010; LMFA02000170; LMFA02000171; LMFA02000172; LMFA02000158; LMFA02000159; LMFA02000286 ; LMFA02000284 ; LMFA02000285 ; LMFA02000115 ;LMFA02000113 ; LMFA02000017; LMFA02000224 |
| 335.2181 | 335.2193 | -3.5 | 0.42 | C18H34O5(Na)(H-2O-1)(H-1) | LMFA02000165 ; LMFA02000169 ; LMFA02000168 ; LMFA02000011; LMFA02000014 ; LMFA02000221 ; LMFA02000222 ; LMFA02000223 |
| 339.2317 | 339.2319 | -0.6 | 0.29 | C23H32O3(H-2O-1) | LMST02010039 |
| 339.2317 | 339.2328 | -3.4 | 0.29 | C18H36O2S(Na)(H-1) | LMFA01130002 |
| 339.2525 | 339.2530 | -1.5 | 0.34 | C20H36O5(H-2O-1) | LMFA03010137 ; LMFA03010069 ; LMFA03010168 ; LMFA03010144 ; LMFA03010079 |
| 339.2525 | 339.2530 | -1.5 | 0.34 | C20H34O4 | LMFA03050016; LMFA03000014 ; LMFA03050006; LMFA03010164 ; LMFA03020004 ; LMFA03050004; LMFA03050008; LMFA03050010; LMFA03050015; LMFA03010054 ; LMFA03010078 |
| 339.2692 | 339.2682 | 2.8 | 1.09 | C24H36O2(H-2O-1) | LMST04010326; LMST04010328; LMST04010442 ; LMST03020688 ; LMST04010388 |
| 339.2692 | 339.2682 | 2.8 | 1.09 | C24H36O2(H-2O-1) | LMFA01030822 ; LMFA01030186; LMFA01030804 |
| 340.2032 | 340.2037 | -1.5 | 0.21 | C20H33NO2(K)(H-2O-1)(H-1) | LMFA08020029 ; LMFA08040006 |
| 340.2271 | 340.2271 | 0 | 0.18 | C22H29NO2 | LMFA08040014 |
| 341.1759 | 341.1747 | 3.3 | 0.27 | C21H26O5(H-2O-1) | LMST02030180 |
| 341.2446 | 341.2451 | -1.6 | 0.47 | C21H34O2(Na)(H-1) | LMST02030173 ; LMST02030130 ; LMST02030175 ; LMST02030185 ; LMST02030206 ; LMST02030205 ; LMST02030157 ; LMST02030156 ; LMST02030155 |
| 341.2697 | 341.2686 | 3.2 | 0.8 | C20H38O5(H-2O-1) | LMFA01080003; LMFA01080005; LMFA01080004; LMFA01050121; LMFA01050122 |
| 341.2697 | 341.2686 | 3.2 | 0.8 | C20H36O4 | LMFA03010113 ; LMFA03010068 ; LMFA01040030 ; LMFA03010053 ; LMFA03010070 |
| 341.2823 | 341.2839 | -4.8 | 0.29 | C24H38O2(H-2O-1) | LMST04010266; LMST04010269; LMST04010268; LMST04010267; LMST04010263; LMST04010265; LMST04010264; LMST04010142; LMST04010143; LMST04010144; LMST04010170 |
| 341.2823 | 341.2839 | -4.8 | 0.29 | C24H38O2(H-2O-1) | LMPR0105060003 |
| 341.2823 | 341.2815 | 2.3 | 0.29 | C22H40O2(Na)(H-2O-1)(H-1) | LMFA01030686; LMFA01030861 ; LMFA01020253; LMFA07010613; LMFA07010401; LMFA01030131; LMFA01030405; LMFA01030406; LMFA04000065 ; LMFA04000061 ; LMFA01030132; LMFA07010399 |
| 341.2823 | 341.2839 | -4.8 | 0.29 | C24H38O2(H-2O-1) | LMFA01030820; LMFA01030821; LMFA01140015 |
| 342.2395 | 342.2404 | -2.5 | 0.17 | C20H33NO2(Na)(H-1) | LMFA08020029 ; LMFA08040006 |
| 343.1886 | 343.1880 | 1.9 | 0.66 | C19H30O5(Na)(H-2O-1)(H-1) | LMST02020108 |
| 343.1886 | 343.1880 | 1.9 | 0.66 | C19H28O4(Na)(H-1) | LMST02020081 |
| 343.1886 | 343.1881 | 1.5 | 0.66 | C16H32O5(K)(H-1) | LMFA01050099 ; LMFA01050102 ; LMFA01050101 ; LMFA01050100 |
| 345.2569 | 345.2554 | 4.4 | 0.17 | C21H40O2(K)(H-2O-1)(H-1) | LMPR0103010015 |
| 345.2569 | 345.2554 | 4.4 | 0.17 | C21H40O2(K)(H-2O-1)(H-1) | LMFA01030398; LMFA01020135; LMFA01020136; LMFA01030909 ; LMFA07010635; LMFA07010671 |
| 345.2569 | 345.2554 | 4.4 | 0.17 | C21H38O(K)(H-1) | LMFA12000213; LMFA12000216; LMFA12000217; LMFA12000218; LMFA12000320; LMFA12000321; LMFA12000322; LMFA12000323; LMFA12000324; LMFA12000325 |
| 346.2758 | 346.2741 | 4.9 | 0.19 | C22H37NO3(H-2O-1) | LMFA03020011 ; LMFA08040039 ; LMFA08040033 ; LMFA08040032 ; LMFA08040035 ; LMFA08040034 ; LMFA08040040 |
| 346.2758 | 346.2741 | 4.9 | 0.19 | C22H35NO2 | LMFA08040008 |
| 347.2214 | 347.2217 | -0.9 | 0.47 | C21H30O4 | LMST02030086 ; LMST02030186 ; LMST03020004 ; LMST02030171; LMST02030196; LMST02030195 |
| 347.2214 | 347.2217 | -0.9 | 0.47 | C21H32O5(H-2O-1) | LMST02030098 ; LMST02030204 ; LMST02030197; LMST02030222 ; LMST02030166 |
| 347.2694 | 347.2711 | -4.8 | 0.16 | C21H42O2(K)(H-2O-1)(H-1) | LMPR02020064 |
| 347.2694 | 347.2711 | -4.8 | 0.16 | C21H42O2(K)(H-2O-1)(H-1) | LMFA07010453; LMFA07010416; LMFA07010576; LMFA01020356 ; LMFA07010556; LMFA01020033; LMFA01020018; LMFA01010021; LMFA07010443; LMFA07010649; LMFA07010509; LMFA01020223 |
| 347.2694 | 347.2711 | -4.8 | 0.16 | C21H40O(K)(H-1) | LMFA12000315; LMFA12000211; LMFA12000214; LMFA12000215; LMFA05000533; LMFA05000534 |
| 347.2943 | 347.2945 | -0.4 | 0.2 | C23H40O3(H-2O-1) | LMGL01010029 ; LMGL01020026 |
| 347.2943 | 347.2945 | -0.4 | 0.2 | C23H40O3(H-2O-1) | LMST04060011; LMST04060010; LMST04060008; LMST04060007; LMST04060009 |
| 347.2943 | 347.2945 | -0.4 | 0.2 | C23H38O2 | LMST04060014; LMST04060016; LMST04060013; LMST04060015 |
| 348.2147 | 348.2145 | 0.5 | 0.26 | C18H31NO4(Na)(H-1) | LMFA01120002; LMFA01120001; LMFA08030007 |
| 349.2128 | 349.2138 | -2.8 | 0.31 | C22H32O3(Na)(H-2O-1)(H-1) | LMST02030176 ; LMST03020007 ; LMST02020076 |
| 349.2128 | 349.2138 | -2.8 | 0.31 | C22H32O3(Na)(H-2O-1)(H-1) | LMFA04000072 ; LMFA04000059 ; LMFA04000058 ; LMFA04000033 ; LMFA04000032 ; LMFA04000031 ; LMFA04000030 ; LMFA04000037 ; LMFA04000036 ; LMFA04000035 ; LMFA04000034 ; LMFA04000038 ; LMFA04000012 ; LMFA03010163 ; LMFA04000060; LMFA04000024 ; LMFA04000025 ; LMFA04000026 ; LMFA04000027 ; LMFA04000028 ; LMFA04000029 |
| 349.2334 | 349.2349 | -4.4 | 0.74 | C19H34O4(Na)(H-1) | LMFA01080002; LMFA01080007; LMFA01170126 |
| 349.2334 | 349.2349 | -4.4 | 0.74 | C19H36O5(Na)(H-2O-1)(H-1) | LMFA01080006 |
| 349.2380 | 349.2373 | 1.8 | 0.19 | C21H32O4 | LMST02030199; LMST02030188; LMST02030140 ; LMST02030141 |
| 349.2380 | 349.2373 | 1.8 | 0.19 | C21H34O5(H-2O-1) | LMST02030139 ; LMST02030143 ; LMST02030200 |
| 349.2380 | 349.2373 | 1.8 | 0.19 | C21H34O5(H-2O-1) | LMFA03010095 ; LMFA03010063 ; LMFA03010064 ; LMFA03010105 ; LMFA03010050 |
| 349.2380 | 349.2373 | 1.8 | 0.19 | C21H32O4 | LMFA03010104 ; LMFA03070015; LMFA03070014; LMFA03070017; LMFA03070016 |
| 350.2314 | 350.2302 | 3.4 | 1.11 | C18H33NO4(Na)(H-1) | LMFA01120003; LMFA01120004 |
| 351.1560 | 351.1567 | -2 | 0.41 | C15H29O8P(H-2O-1) | LMGP10010020 |
| 351.1560 | 351.1567 | -1.9 | 0.41 | C20H24O4(Na)(H-1) | LMPR0104010020 |
| 351.1560 | 351.1567 | -1.9 | 0.41 | C20H26O5(Na)(H-2O-1)(H-1) | LMPR0104170018 ; LMPR0104170030 |
| 351.2328 | 351.2319 | 2.6 | 0.22 | C24H32O3(H-2O-1) | LMST04010429 |
| 351.3255 | 351.3258 | -0.7 | 0.76 | C23H42O2 | LMPR0103010018 |
| 351.3255 | 351.3258 | -0.7 | 0.76 | C23H44O3(H-2O-1) | LMFA01060144; LMFA01060145; LMFA01060146 |
| 352.2261 | 352.2247 | 3.7 | 0.16 | C16H34NO5P | LMSP01050005 |
| 352.2261 | 352.2249 | 3.4 | 0.16 | C18H35NO3(K)(H-1) | LMFA08020079 |
| 352.2386 | 352.2401 | -4.2 | 0.38 | C22H37NO(K)(H-2O-1)(H-1) | LMFA08020006 ; LMFA08020023 |
| 353.2234 | 353.2241 | -2 | 0.18 | C22H36O2(K)(H-2O-1)(H-1) | LMFA04000050 ; LMFA07010351; LMFA01140012; LMFA01030178 ; LMFA01030179; LMFA01030177; LMFA04000066 ; LMFA04000063 |
| 353.2443 | 353.2451 | -2.3 | 0.32 | C22H34O2(Na)(H-1) | LMST03020010 ; LMST03020009 |
| 353.2443 | 353.2451 | -2.3 | 0.32 | C22H34O2(Na)(H-1) | LMPR0104390004 |
| 353.2443 | 353.2453 | -2.8 | 0.32 | C19H38O3(K)(H-1) | LMFA01050072; LMFA01050071 |
| 353.2443 | 353.2451 | -2.3 | 0.32 | C22H34O2(Na)(H-1) | LMFA01140013; LMFA01030183; LMFA01030182; LMFA01030184; LMFA04000064 ; LMFA04000049 ; LMFA04000044 |
| 353.2489 | 353.2475 | 4 | 0.63 | C24H34O3(H-2O-1) | LMST04010341; LMST04010235; LMST04010333; LMST04010332; LMST04010331; LMST04010318; LMST04010234 |
| 353.2613 | 353.2605 | 2.3 | 0.38 | C23H38(K)(H-1) | LMFA11000180 |
| 354.2420 | 354.2404 | 4.4 | 0.52 | C16H36NO5P | LMSP01050006 |
| 354.2420 | 354.2405 | 4.1 | 0.52 | C18H37NO3(K)(H-1) | LMSP01030002 ; LMSP01080003 ; LMSP01080009 |
| 354.2578 | 354.2567 | 3.1 | 0.16 | C22H36FNO(Na)(H-2O-1)(H-1) | LMFA08020055 |
| 355.1792 | 355.1801 | -2.6 | 1.5 | C18H35ClO3(K)(H-2O-1)(H-1) | LMFA01090058; LMFA01090055; LMFA01090057; LMFA01090056 |
| 355.2227 | 355.2244 | -4.8 | 0.17 | C21H34O4(Na)(H-2O-1)(H-1) | LMST05030014 ; LMST02030100 ; LMST02030142 |
| 355.2227 | 355.2244 | -4.8 | 0.17 | C21H32O3(Na)(H-1) | LMST02030172 ; LMST02030134 ; LMST02030089 ; LMST02030183 ; LMST03020006 ; LMST02030208; LMST02030149 ; LMST02030151 ;LMST02030167 |
| 355.2227 | 355.2244 | -4.8 | 0.17 | C21H32O3(Na)(H-1) | LMPR0104270001 |
| 355.2227 | 355.2244 | -4.8 | 0.17 | C21H34O4(Na)(H-2O-1)(H-1) | LMPR0104360001 |
| 355.2227 | 355.2244 | -4.8 | 0.17 | C21H34O4(Na)(H-2O-1)(H-1) | LMFA03010103 ; LMFA03010058 |
| 355.2476 | 355.2479 | -0.8 | 0.74 | C20H34O5 | LMPR0104210002 |
| 355.2476 | 355.2479 | -0.8 | 0.74 | C20H34O5 | LMFA03010150 ; LMFA03010134 ; LMFA03050017; LMFA03050018; LMFA03110164 ; LMFA03110165 ; LMFA03110166 ; LMFA03110160 ; LMFA03110161 ; LMFA03110162 ; LMFA03110163 ; LMFA03110148 ; LMFA03110149 ; LMFA03110143 ;LMFA03110146 ; LMFA03110147 ; LMFA03110144 ; LMFA03110145 ; LMFA03110108 ; LMFA03110109 ;LMFA03010049 ; LMFA03010044 ; LMFA03010041 ; LMFA03010002 ; LMFA03010027 ; LMFA03010025 ; LMFA03110006 ; LMFA03110004; LMFA03110002 ; LMFA03110001 ; LMFA03110029 ; LMFA03110028 ; LMFA03110025 ; LMFA03110024 ; LMFA03110027; LMFA03110026 ; LMFA03110021 ; LMFA03110020 ; LMFA03110023 ; LMFA03110022 ; LMFA03110048 ;LMFA03110043 ; LMFA03110042 ; LMFA03110041 ; LMFA03110040 ; LMFA03110047 ; LMFA03110046 ; LMFA03110045 ; LMFA03110044 ; LMFA03010145 ; LMFA03010129 ; LMFA03010128 ; LMFA03010106 ; LMFA03110155 ; LMFA03110154; LMFA03110157 ; LMFA03110156 ; LMFA03110151 ; LMFA03110150 ; LMFA03110153 ; LMFA03110152 ; LMFA03110159 ; LMFA03110158 ; LMFA03110111 ; LMFA03110110 ; LMFA03110113 ; LMFA03110112 ; LMFA03090002 ; LMFA03090004 ; LMFA03010055 ; LMFA03010203 ; LMFA03010077 ; LMFA03110011  5; LMFA03110018; LMFA03110019 ; LMFA03010036 ; LMFA03110032 ; LMFA03110033 ; LMFA03110030 ; LMFA03110031 ; LMFA03110036 ; LMFA03110037 ; LMFA03110034 ; LMFA03110035 ; LMFA03110038 ; LMFA03110039 |
| 355.2476 | 355.2479 | -0.8 | 0.74 | C20H36O6(H-2O-1) | LMFA03030008 ; LMFA03010165 ; LMFA03010038 |
| 355.2603 | 355.2608 | -1.1 | 0.29 | C22H38O3(Na)(H-2O-1)(H-1) | LMFA01150002 |
| 355.2603 | 355.2608 | -1.1 | 0.29 | C22H36O2(Na)(H-1) | LMFA04000050 ; LMFA07010351; LMFA01140012; LMFA01030178 ; LMFA01030179; LMFA01030177; LMFA04000066 ; LMFA04000063 |
| 357.1733 | 357.1722 | 2.8 | 0.17 | C18H34Cl2O2(Na)(H-2O-1)(H-1) | LMFA01090059 |
| 359.1843 | 359.1853 | -2.7 | 0.33 | C21H26O5 | LMST02030180 |
| 359.1843 | 359.1853 | -2.7 | 0.33 | C21H28O6(H-2O-1) | LMST02030194 |
| 359.1843 | 359.1829 | 4 | 0.33 | C19H30O6(Na)(H-2O-1)(H-1) | LMFA01040028 |
| 359.2338 | 359.2347 | -2.6 | 0.24 | C21H36O2(K)(H-1) | LMST02030202 ; LMST02020100; LMST02030198 |
| 359.2338 | 359.2345 | -2.2 | 0.24 | C24H34O2(Na)(H-2O-1)(H-1) | LMST04010327 |
| 359.2338 | 359.2345 | -2.2 | 0.24 | C24H34O2(Na)(H-2O-1)(H-1) | LMFA01030852 |
| 359.2338 | 359.2347 | -2.6 | 0.24 | C21H36O2(K)(H-1) | LMFA01030864 ; LMFA12000212; LMFA07010545 |
| 361.1872 | 361.1857 | 4.2 | 0.65 | C17H28O8 | LMFA13010039 |
| 361.1996 | 361.1985 | 2.9 | 0.3 | C19H30O5(Na)(H-1) | LMST02020108 |
| 361.1996 | 361.2010 | -3.8 | 0.3 | C21H30O6(H-2O-1) | LMST02030193 |
| 361.1996 | 361.2010 | -3.8 | 0.3 | C21H28O5 | LMST02030179 ; LMST02030090 ; LMST02030145 ; LMST02030026 |
| 361.1996 | 361.1985 | 2.9 | 0.3 | C19H32O6(Na)(H-2O-1)(H-1) | LMFA01040049; LMFA01040048; LMFA01040043; LMFA01040042; LMFA01040041; LMFA01040040; LMFA01040047; LMFA01040046; LMFA01040045; LMFA01040044; LMFA01040050; LMFA01040053; LMFA01040055; LMFA01040056 |
| 361.2122 | 361.2138 | -4.3 | 2.18 | C23H32O3(Na)(H-2O-1)(H-1) | LMST02010039 |
| 361.2122 | 361.2140 | -4.7 | 2.18 | C20H34O3(K)(H-1) | LMPR0104240002 ; LMPR0104110002 ; LMPR0104150001 |
| 361.2122 | 361.2140 | -4.7 | 2.18 | C20H34O3(K)(H-1) | LMFA01060073; LMFA03050011 ; LMFA03050007 ; LMFA03050005 ; LMFA03050012 ; LMFA01150003 |
| 361.2122 | 361.2140 | -4.7 | 2.18 | C20H36O4(K)(H-2O-1)(H-1) | LMFA03010113 ; LMFA03010068 ; LMFA01040030 ; LMFA03010053 ; LMFA03010070 |
| 363.1978 | 363.1966 | 3.2 | 0.7 | C21H29FO5(H-2O-1) | LMST02030103 |
| 363.2102 | 363.2085 | 4.8 | 1.04 | C23H32O(K)(H-1) | LMPR01090045 |
| 363.2102 | 363.2085 | 4.8 | 1.04 | C23H34O2(K)(H-2O-1)(H-1) | LMFA01030853 |
| 364.1902 | 364.1885 | 4.7 | 0.23 | C18H31NO4(K)(H-1) | LMFA01120002; LMFA01120001; LMFA08030007 |
| 364.2238 | 364.2249 | -2.9 | 0.5 | C19H37NO4(K)(H-2O-1)(H-1) | LMFA08020101 |
| 365.1954 | 365.1959 | -1.2 | 0.37 | C20H28O6 | LMPR0104170016 ; LMPR0104170008 ; LMPR0104330001 ; LMPR0104040002 |
| 365.2081 | 365.2088 | -1.7 | 0.77 | C17H35O7P(H-2O-1) | LMGP10050007 |
| 365.2081 | 365.2087 | -1.6 | 0.77 | C22H30O3(Na)(H-1) | LMPR0104410001 |
| 365.2081 | 365.2089 | -2 | 0.77 | C19H34O4(K)(H-1) | LMFA01080002; LMFA01080007; LMFA01170126 |
| 365.2081 | 365.2089 | -2 | 0.77 | C19H36O5(K)(H-2O-1)(H-1) | LMFA01080006 |
| 365.2081 | 365.2087 | -1.6 | 0.77 | C22H30O3(Na)(H-1) | LMFA04000052 ; LMFA04000014 |
| 365.2081 | 365.2087 | -1.6 | 0.77 | C22H32O4(Na)(H-2O-1)(H-1) | LMFA04000071 ; LMFA04000055 ; LMFA04000054 ; LMFA04000057 ; LMFA04000056 ; LMFA04000051 ; LMFA04000011 ;LMFA04000010 ; LMFA01020290; LMFA04000069 ; LMFA04000067 ; LMFA04000048 ; LMFA04000047 ; LMFA04000045 |
| 365.2331 | 365.2323 | 2.4 | 0.4 | C21H32O5 | LMST02030098 ; LMST02030204 ; LMST02030197; LMST02030222 ; LMST02030166 |
| 366.1447 | 366.1442 | 1.3 | 0.76 | C13H30NO6P(K)(H-1) | LMGP01060020 |
| 367.1370 | 367.1363 | 1.8 | 0.49 | C16H24O8(Na)(H-1) | LMPR0102070023 ; LMPR0102070024 |
| 367.1451 | 367.1437 | 4 | 0.22 | C18H29ClO3(K)(H-1) | LMFA03120053 |
| 368.2233 | 368.2230 | 1 | 0.19 | C18H37NO4S(Na)(H-2O-1)(H-1) | LMFA08020080 |
| 368.2358 | 368.2350 | 2 | 0.33 | C22H37NO2(K)(H-2O-1)(H-1) | LMFA00000014 ; LMFA08040001 |
| 369.1472 | 369.1463 | 2.5 | 0.6 | C20H28O5(K)(H-2O-1)(H-1) | LMPR0104170017 ; LMPR0104170015 ; LMPR0104420001 ; LMPR0104170007 |
| 369.1472 | 369.1463 | 2.5 | 0.6 | C20H28O5(K)(H-2O-1)(H-1) | LMFA03070047 ; LMFA03070044 |
| 369.2281 | 369.2272 | 2.6 | 0.34 | C20H32O6 | LMPR0104180003 |
| 369.2281 | 369.2272 | 2.6 | 0.34 | C20H32O6 | LMFA03060098 ; LMFA03060097 ; LMFA03030006 ; LMFA03030004 ; LMFA03010217 ; LMFA03010216 ; LMFA03010009 ;LMFA03010024 ; LMFA03010149 ; LMFA03010220 ; LMFA03010013 ; LMFA03010012 ; LMFA03010014 ;LMFA03060048 ; LMFA03060047 ; LMFA03060046 |
| 372.8689 | 372.8690 | -0.3 | 0.28 | C10H11BrCl4(Na)(H-1) | LMPR0102030001 |
| 373.1747 | 373.1750 | -1 | 0.48 | C16H33O7P(Na)(H-2O-1)(H-1) | LMGP10050001 |
| 374.1709 | 374.1703 | 1.5 | 0.51 | C15H32NO7P(Na)(H-2O-1)(H-1) | LMGP01050064 ; LMGP01050095 |
| 375.1632 | 375.1650 | -4.8 | 0.47 | C17H26O9 | LMPR0102070005 ; LMPR0102070020 |
| 375.1839 | 375.1828 | 2.8 | 0.38 | C18H34Cl2O2(Na)(H-1) | LMFA01090059 |
| 377.1721 | 377.1725 | -1 | 0.72 | C19H30O5(K)(H-1) | LMST02020108 |
| 377.1721 | 377.1723 | -0.6 | 0.72 | C22H28O5(Na)(H-2O-1)(H-1) | LMPR0102060022 ; LMPR0104320001 |
| 377.1721 | 377.1725 | -1 | 0.72 | C19H32O6(K)(H-2O-1)(H-1) | LMFA01040049; LMFA01040048; LMFA01040043; LMFA01040042; LMFA01040041; LMFA01040040; LMFA01040047; LMFA01040046; LMFA01040045; LMFA01040044; LMFA01040050; LMFA01040053; LMFA01040055; LMFA01040056 |
| 377.1721 | 377.1723 | -0.6 | 0.72 | C22H28O5(Na)(H-2O-1)(H-1) | LMFA03010066 ; LMFA03120017 ; LMFA03120016 ; LMFA03120015 |
| 377.1927 | 377.1935 | -2 | 0.2 | C19H30O6(Na)(H-1) | LMFA01040028 |
| 377.1969 | 377.1959 | 2.8 | 0.28 | C21H28O6 | LMST02030194 |
| 378.1639 | 378.1652 | -3.5 | 0.37 | C14H30NO7P(Na)(H-1) | LMGP01050062 ; LMGP01050093 |
| 379.1682 | 379.1670 | 3.3 | 0.17 | C22H30O4(K)(H-2O-1)(H-1) | LMST02030154 |
| 379.1682 | 379.1671 | 3.1 | 0.17 | C21H29ClO5(H-2O-1) | LMFA03120012 |
| 379.1682 | 379.1670 | 3.3 | 0.17 | C22H30O4(K)(H-2O-1)(H-1) | LMFA04000073 ; LMFA04000070 ; LMFA04000013 ; LMFA04000001 |
| 379.1889 | 379.1880 | 2.4 | 0.16 | C22H30O5(Na)(H-2O-1)(H-1) | LMST02030178 ; LMST02030190; LMST02030158 |
| 379.1889 | 379.1880 | 2.4 | 0.16 | C22H30O5(Na)(H-2O-1)(H-1) | LMPR0102060005 |
| 379.1889 | 379.1880 | 2.4 | 0.16 | C22H30O5(Na)(H-2O-1)(H-1) | LMFA04000080 ; LMFA04000079 |
| 379.1934 | 379.1938 | -0.8 | 0.21 | C21H32O5S(H-2O-1) | LMST05020014 |
| 381.1893 | 381.1884 | 2.5 | 0.24 | C18H32O8(Na)(H-2O-1)(H-1) | LMSL05000001 |
| 383.1979 | 383.1983 | -1.1 | 0.19 | C22H32O3(K)(H-1) | LMST02030176 ; LMST03020007 ; LMST02020076 |
| 383.1979 | 383.1983 | -1.1 | 0.19 | C22H34O4(K)(H-2O-1)(H-1) | LMFA03010158 ; LMFA03010159 ; LMFA04000039 ; LMFA03010086 ; LMFA04000042 ; LMFA04000043 ;LMFA04000040 ; LMFA04000041 |
| 383.1979 | 383.1983 | -1.1 | 0.19 | C22H32O3(K)(H-1) | LMFA04000072 ; LMFA04000059 ; LMFA04000058 ; LMFA04000033 ; LMFA04000032 ; LMFA04000031 ; LMFA04000030 ; LMFA04000037 ; LMFA04000036 ; LMFA04000035 ; LMFA04000034 ; LMFA04000038 ; LMFA04000012 ; LMFA03010163 ; LMFA04000060; LMFA04000024 ; LMFA04000025 ; LMFA04000026 ; LMFA04000027 ; LMFA04000028 ; LMFA04000029 |
| 385.1121 | 385.1105 | 4.2 | 0.36 | C15H22O10(Na)(H-1) | LMPR0102070007 |
| 385.1246 | 385.1258 | -3 | 0.18 | C19H22O7(Na)(H-1) | LMPR0104170027 |
| 387.1201 | 387.1204 | -0.8 | 0.54 | C19H24O6(K)(H-1) | LMST02020063 |
| 387.1201 | 387.1204 | -0.8 | 0.54 | C19H24O6(K)(H-1) | LMPR0104170025 ; LMPR0104170036 ; LMPR0104170001 |
| 389.1358 | 389.1361 | -0.6 | 0.26 | C19H26O6(K)(H-1) | LMPR0103090013 ; LMPR0104070003 |
| 389.1402 | 389.1393 | 2.3 | 0.63 | C19H28O6S(Na)(H-2O-1)(H-1) | LMST05020022 |
| 390.1438 | 390.1442 | -1.2 | 0.41 | C15H32NO7P(K)(H-2O-1)(H-1) | LMGP01050064 ; LMGP01050095 |
| 391.1226 | 391.1235 | -2.2 | 0.22 | C16H22O11 | LMPR0102070012 |
| 391.1226 | 391.1243 | -4.3 | 0.22 | C19H29BrO2(Na)(H-1) | LMFA01090086 ; LMFA01090087 |
| 391.1349 | 391.1340 | 2.3 | 0.56 | C19H30O5S(K)(H-2O-1)(H-1) | LMST05020023 ; LMST05020001 |
| 391.1474 | 391.1492 | -4.8 | 0.22 | C15H29O8P(Na)(H-1) | LMGP10010020 |
| 393.1421 | 393.1439 | -4.6 | 0.32 | C15H31O7P(K)(H-1) | LMGP10050015 |
| 395.0758 | 395.0739 | 4.8 | 0.18 | C16H20O9(K)(H-1) | LMPR0102070009 |
| 396.0876 | 396.0873 | 0.6 | 0.19 | C18H21O9S(H-2O-1) | LMFA07060033 |
| 398.1015 | 398.1030 | -3.7 | 0.23 | C18H21O8S | LMFA07060023 |
| 399.1210 | 399.1204 | 1.3 | 0.45 | C20H26O7(K)(H-2O-1)(H-1) | LMPR0104170034 |
| 400.1197 | 400.1186 | 2.8 | 0.67 | C18H23O8S | LMFA07060032 |
| 401.1268 | 401.1280 | -3.1 | 0.55 | C21H29ClO4(K)(H-2O-1)(H-1) | LMFA03120008 ; LMFA03120009 ; LMFA03120011 ; LMFA03120010 |
| 401.1350 | 401.1361 | -2.8 | 0.27 | C20H26O6(K)(H-1) | LMPR0104170019 ; LMPR0104170039 |
| 403.1160 | 403.1154 | 1.7 | 0.18 | C19H24O7(K)(H-1) | LMPR0104170005 |
| 404.9475 | 404.9460 | 3.8 | 0.5 | C16H18Br2O2(Na)(H-2O-1)(H-1) | LMFA01090115 |
| 406.0686 | 406.0693 | -1.6 | 0.75 | C17H19O8S(Na)(H-1) | LMFA07060022 |
| 408.0832 | 408.0849 | -4.4 | 0.86 | C17H21O8S(Na)(H-1) | LMFA07060031 |
| 409.0900 | 409.0895 | 1.1 | 0.42 | C17H24O10(K)(H-2O-1)(H-1) | LMPR0102070002 ; LMPR0102070016 |
| 411.0926 | 411.0932 | -1.4 | 0.41 | C18H29BrO3(K)(H-1) | LMFA01090094 ; LMFA01090093 |
| 411.1049 | 411.1052 | -0.7 | 0.55 | C17H26O10(K)(H-2O-1)(H-1) | LMPR0102070001 |
| 413.1075 | 413.1054 | 4.9 | 0.44 | C16H22O11(Na)(H-1) | LMPR0102070012 |
| 413.1197 | 413.1207 | -2.3 | 0.64 | C20H24O9(Na)(H-2O-1)(H-1) | LMPR0104540001 |
| 413.1197 | 413.1208 | -2.6 | 0.64 | C17H26O9(K)(H-1) | LMPR0102070005 ; LMPR0102070020 |
| 413.1326 | 413.1336 | -2.5 | 0.59 | C20H31IO2(H-2O-1) | LMFA03000015 |
| 413.2659 | 413.2662 | -0.7 | 0.81 | C24H40O5(Na)(H-2O-1)(H-1) | LMST04010417; LMST04010414; LMST04010411 ; LMST04010418; LMST04010108; LMST04010339; LMST04010300; LMST04010086; LMST04010083; LMST04010001 ; LMST04010250; LMST04010256;LMST04010257; LMST04010070; LMST04010071; LMST04010072; LMST04010073; LMST04010074; LMST04010075; LMST04010076; LMST04010077; LMST04010078; LMST04010079; LMST04010109; LMST04010104; LMST04010105;LMST04010107 ; LMST04010100; LMST04010101 ; LMST04010102 ; LMST04010103; LMST04010249; LMST04010248; LMST04010247; LMST04010246; LMST04010063; LMST04010062;LMST04010061; LMST04010060; LMST04010067 ; LMST04010066 ; LMST04010065 ; LMST04010064 ; LMST04010069; LMST04010068; LMST04010113; LMST04010112; LMST04010111; LMST04010110; LMST04010058; LMST04010057 ; LMST04010285; LMST04010251; LMST04010089; LMST04010088 ; LMST04010059; LMST04010449; LMST04010092 ; LMST04010093; LMST04010090; LMST04010091; LMST04010096; LMST04010097; LMST04010094; LMST04010095; LMST04010098; LMST04010099; LMST04010085; LMST04010087;LMST04010081; LMST04010080; LMST04010082; LMST04010373; LMST04010372; LMST04010371; LMST04010370 |
| 413.2659 | 413.2662 | -0.7 | 0.81 | C24H38O4(Na)(H-1) | LMST04010347; LMST04010404; LMST04010214; LMST04010216; LMST04010217; LMST04010218; LMST04010219; LMST04010228; LMST04010230; LMST04010226; LMST04010229; LMST04010225; LMST04010224; LMST04010221;LMST04010220; LMST04010223; LMST04010222; LMST04010258; LMST04010279; LMST04010298; LMST04010284; LMST04010280; LMST04010146; LMST04010147; LMST04010148; LMST04010149; LMST04010153; LMST04010152;LMST04010151; LMST04010150; LMST04010157; LMST04010156; LMST04010155; LMST04010154; LMST04010159; LMST04010158; LMST04010438; LMST04010385; LMST04010168; LMST04010169; LMST04010166; LMST04010167;LMST04010164; LMST04010165; LMST04010162; LMST04010163; LMST04010160; LMST04010161; LMST04010367; LMST04010426; LMST04010395; LMST04010398; LMST04010171 |
| 415.1475 | 415.1468 | 1.6 | 0.21 | C18H35IO2(Na)(H-2O-1)(H-1) | LMFA01090040 |
| 419.2536 | 419.2557 | -5 | 0.41 | C21H41O7P(H-2O-1) | LMGP10050014 ; LMGP10050008 |
| 419.2536 | 419.2533 | 0.7 | 0.41 | C19H41O6P(Na)(H-1) | LMGP10060005 |
| 419.2536 | 419.2557 | -5 | 0.41 | C21H39O6P | LMGP00000056 |
| 420.0660 | 420.0640 | 4.8 | 0.19 | C18H23O8S(K)(H-2O-1)(H-1) | LMFA07060032 |
| 421.0945 | 421.0942 | 0.7 | 0.29 | C15H28O7P2(K)(H-1) | LMPR0103010009 ; LMPR0103010002 ; LMPR0103010003 ; LMPR0103010011 ; LMPR0103010010 |
| 423.0888 | 423.0893 | -1.2 | 0.17 | C18H34Br2O2(H-2O-1) | LMFA01090084 |
| 426.0995 | 426.0979 | 3.6 | 0.28 | C19H21O9S | LMFA07060025 |
| 428.1149 | 428.1136 | 3.2 | 0.2 | C19H23O9S | LMFA07060034 |
| 429.1351 | 429.1367 | -3.9 | 0.3 | C17H26O11(Na)(H-1) | LMPR0102070028 |
| 429.2366 | 429.2376 | -2.4 | 0.79 | C20H41O7P(Na)(H-2O-1)(H-1) | LMGP10050036 |
| 429.2366 | 429.2363 | 0.8 | 0.79 | C23H41BrO2 | LMFA01090101 |
| 429.3729 | 429.3727 | 0.5 | 0.25 | C29H48O2 | LMST01040167 ; LMST01040169; LMST03020416 ; LMST03020417 ; LMST03020415 ; LMST03020418 ; LMST03020673 ; LMST01040196 ; LMST01040168 ; LMST03040004 ; LMST01010232;LMST01010230; LMST01010204 ; LMST01040151 ; LMST01110017; LMST01010245 |
| 429.3729 | 429.3727 | 0.5 | 0.25 | C29H50O3(H-2O-1) | LMST01040188; LMST01040177 ; LMST01031113 |
| 430.0700 | 430.0693 | 1.6 | 0.24 | C19H21O9S(Na)(H-2O-1)(H-1) | LMFA07060025 |
| 432.0852 | 432.0849 | 0.5 | 0.18 | C19H23O9S(Na)(H-2O-1)(H-1) | LMFA07060034 |
| 435.1136 | 435.1155 | -4.4 | 0.7 | C20H31IO2(Na)(H-2O-1)(H-1) | LMFA03000015 |
| 441.2950 | 441.2930 | 4.7 | 5.19 | C27H43FO(K)(H-1) | LMST03020663 ; LMST03020199 ; LMST03020198 ; LMST03020197 ; LMST03020202 ; LMST03020201 ; LMST03020200 |
| 441.2950 | 441.2939 | 2.4 | 5.19 | C27H42F2O2(Na)(H-2O-1)(H-1) | LMST03020140 ; LMST03020141 ; LMST03020139 ; LMST03020138 ; LMST03020137 |
| 443.2515 | 443.2533 | -4 | 0.18 | C21H41O6P(Na)(H-1) | LMGP00000055 |
| 443.2515 | 443.2533 | -4 | 0.18 | C21H43O7P(Na)(H-2O-1)(H-1) | LMGP10050005 |
| 443.2515 | 443.2519 | -0.9 | 0.18 | C24H43BrO2 | LMFA01090102 |
| 445.0874 | 445.0895 | -4.9 | 0.34 | C20H24O10(K)(H-2O-1)(H-1) | LMPR0104540004 ; LMPR0104540002 |
| 447.1037 | 447.1052 | -3.3 | 0.42 | C20H24O9(K)(H-1) | LMPR0104540001 |
| 448.9507 | 448.9512 | -1.3 | 0.52 | C18H22Br2O2(K)(H-2O-1)(H-1) | LMFA01090120 |
| 455.0975 | 455.0983 | -1.7 | 0.98 | C23H31BrO3(K)(H-2O-1)(H-1) | LMFA01090110 |
| 457.1141 | 457.1139 | 0.3 | 0.59 | C23H31BrO2(K)(H-1) | LMFA01090107 |
| 457.2744 | 457.2747 | -0.5 | 6.28 | C26H42O3S(Na)(H-1) | LMST03020052 ; LMST03020053 ; LMST03020050 ; LMST03020051 ; LMST03020054 ; LMST03020055 ; LMST03020049 |
| 458.2782 | 458.2780 | 0.5 | 1.59 | C24H41N3O3(K)(H-1) | LMFA08020133 |
| 459.2520 | 459.2506 | 3.1 | 0.49 | C28H38O5(Na)(H-2O-1)(H-1) | LMST01160006 |
| 459.2520 | 459.2540 | -4.3 | 0.49 | C25H40O4S(Na)(H-1) | LMST03020031 |
| 459.2520 | 459.2506 | 3 | 0.49 | C23H39O7P | LMGP10050013 |
| 459.2520 | 459.2507 | 2.7 | 0.49 | C25H42O6(K)(H-2O-1)(H-1) | LMPR0104150002 |
| 459.2737 | 459.2717 | 4.4 | 0.34 | C25H40O6(Na)(H-1) | LMPR0105040002 |
| 459.2822 | 459.2835 | -3 | 0.21 | C27H42F2O(K)(H-1) | LMST03020667 ; LMST03020135 ; LMST03020134 ; LMST03020136 |
| 473.1179 | 473.1183 | -0.9 | 0.25 | C21H29IO4 | LMFA03120014 |
| 473.2776 | 473.2791 | -3.1 | 0.55 | C26H43O4P(Na)(H-1) | LMST03020072 |
| 473.2776 | 473.2793 | -3.5 | 0.55 | C27H43ClO3(Na)(H-1) | LMST03020196 ; LMST03020601 |
| 473.2776 | 473.2793 | -3.4 | 0.55 | C23H49O6P(K)(H-2O-1)(H-1) | LMGP10060003 |
| 478.3430 | 478.3446 | -3.2 | 1.05 | C30H51NO2(K)(H-2O-1)(H-1) | LMST03020684 |
| 484.1513 | 484.1494 | 4 | 0.57 | C24H31O9(K)(H-2O-1)(H-1) | LMST05010019 |
| 484.1513 | 484.1497 | 3.4 | 0.57 | C20H32NO8P(K)(H-1) | LMGP01011235 |
| 485.1601 | 485.1606 | -0.9 | 0.47 | C21H34O8S(K)(H-1) | LMST02030212 |
| 487.2987 | 487.2984 | 0.5 | 0.42 | C28H45FO3(K)(H-1) | LMST03020328 ; LMST03020329 |
| 487.2987 | 487.2973 | 2.8 | 0.42 | C31H46O3(K)(H-2O-1)(H-1) | LMST03020480 |
| 487.2987 | 487.2973 | 2.8 | 0.42 | C31H46O3(K)(H-2O-1)(H-1) | LMPR02030031 |
| 487.2987 | 487.2989 | -0.4 | 0.42 | C28H42N2O3S | LMFA08020053 |
| 497.2080 | 497.2065 | 3 | 0.2 | C23H39O7P(K)(H-1) | LMGP10050013 |
| 529.2716 | 529.2691 | 4.8 | 0.22 | C25H47O7P(K)(H-1) | LMGP10050030 |
| 597.4832 | 597.4853 | -3.5 | 1.35 | C37H68O5(Na)(H-2O-1)(H-1) | LMGL02010413 ; LMGL02010390 ; LMGL02010027 ; LMGL02010026 ; LMGL02010021 ;LMGL02010022 ; LMGL02010349 |
| 598.5511 | 598.5534 | -3.7 | 1.25 | C38H75NO3(Na)(H-2O-1)(H-1) | LMSP02010016 ; LMSP02010007 |
| 599.4989 | 599.5010 | -3.5 | 0.89 | C37H70O5(Na)(H-2O-1)(H-1) | LMGL02010389 ; LMGL02010018 ; LMGL02010459 ; LMGL02010412 ; LMGL02010006 ; LMGL02010005; LMGL02010004 ; LMGL02010023 ; LMGL02010307 ; LMGL02010348 |
| 599.5542 | 599.5528 | 2.4 | 0.27 | C39H78O2(K)(H-2O-1)(H-1) | LMFA01020301 ; LMFA07010007 |
| 601.5162 | 601.5190 | -4.7 | 0.33 | C39H70O5(H-2O-1) | LMGL02010056 ; LMGL02010057 ; LMGL02010479 ; LMGL02010526 ; LMGL02010398; LMGL02010061 ; LMGL02010062 ; LMGL02010421 |
| 601.5162 | 601.5166 | -0.7 | 0.33 | C37H72O5(Na)(H-2O-1)(H-1) | LMGL02010388 ; LMGL02010015 ; LMGL02010435 ; LMGL02010370 ; LMGL02010003 ; LMGL02010020 ;LMGL02010347 |
| 603.5326 | 603.5323 | 0.5 | 0.68 | C37H72O4(Na)(H-1) | LMGL02020001 |
| 603.5326 | 603.5347 | -3.5 | 0.68 | C39H72O5(H-2O-1) | LMGL02010525 ; LMGL02010058 ; LMGL02010050 ; LMGL02010054 ; LMGL02010055 ; LMGL02010397 ; LMGL02010049 ; LMGL02010420 |
| 603.5326 | 603.5347 | -3.5 | 0.68 | C39H70O4 | LMGL02070004 |
| 683.5357 | 683.5375 | -2.6 | 0.69 | C42H78O5(K)(H-2O-1)(H-1) | LMGL02010533 ; LMGL02010148 ; LMGL02010129 ; LMGL02010155 ; LMGL02010135 ; LMGL02010134 |
| 683.5357 | 683.5374 | -2.4 | 0.69 | C40H77O7P(H-2O-1) | LMGP10030045 ; LMGP10030065 ; LMGP10020051 |
| 697.5686 | 697.5684 | 0.3 | 0.28 | C47H80O2(K)(H-2O-1)(H-1) | LMST01020012 |
| 703.5424 | 703.5449 | -3.5 | 2.09 | C50H70O2 | LMPR02030027 |
| 713.5542 | 713.5568 | -3.6 | 0.58 | C38H79N2O6P(Na)(H-1) | LMSP03010091 |
| 714.5584 | 714.5568 | 2.2 | 0.26 | C46H73N3O4(H-2O-1) | LMPR04000016 |
| 737.5571 | 737.5562 | 1.2 | 1.18 | C43H78O10(H-2O-1) | LMGL05010026 |
| 738.6246 | 738.6242 | 0.5 | 0.53 | C44H85NO8(H-2O-1) | LMSP0501AA30 ; LMSP0501AA06 ; LMSP0501AC09 ; LMSP0501AC03 |
| 739.5716 | 739.5719 | -0.4 | 0.63 | C43H80O10(H-2O-1) | LMGL05010025 |
| 739.5716 | 739.5724 | -1.2 | 0.63 | C40H81N2O6P(Na)(H-1) | LMSP03010044 ; LMSP03010045 |
| 796.4896 | 796.4889 | 0.8 | 0.29 | C41H78NO10P(K)(H-2O-1)(H-1) | LMGP03010327 ; LMGP03010133 ; LMGP03010513 ; LMGP03010532 ; LMGP03010458 ; LMGP03010089 ; LMGP03010258; LMGP03010214 ; LMGP03010180 ; LMGP03010726 ; LMGP03010893 ; LMGP03010486 ; LMGP03010313 ;LMGP03010677 ; LMGP03010196 ; LMGP03010151 |
| 796.4896 | 796.4888 | 1 | 0.29 | C44H74NO9P(Na)(H-2O-1)(H-1) | LMGP03030092 |
| 796.4896 | 796.4888 | 1 | 0.29 | C44H72NO8P(Na)(H-1) | LMGP01010964 ; LMGP01010961 ; LMGP01011716 ; LMGP02011141; LMGP02010620 |
| 796.4896 | 796.4889 | 0.8 | 0.29 | C41H76NO9P(K)(H-1) | LMGP03030038 |
| 850.6529 | 850.6533 | -0.4 | 1.08 | C48H93NO8(K)(H-1) | LMSP0501AA22 ; LMSP0501AA09 ; LMSP0501AC05 |
| 852.6499 | 852.6477 | 2.6 | 0.44 | C49H90NO8P | LMGP01012083 ; LMGP01011920 ; LMGP02011128 ; LMGP01011754 ; LMGP02011038 ;LMGP02011099 ; LMGP01011974 |
| 894.6693 | 894.6712 | -2.1 | 0.37 | C50H98NO7P(K)(H-1) | LMGP01030101 ; LMGP01020242 |
| 894.6693 | 894.6712 | -2.1 | 0.37 | C50H100NO8P(K)(H-2O-1)(H-1) | LMGP01011994 ; LMGP01011025 ; LMGP01011150 ; LMGP01010663 ; LMGP01010825 ; LMGP01011072 |
| 1026.7630 | 1026.7652 | -2.1 | 0.39 | C60H112NO8P(K)(H-2O-1)(H-1) | LMGP01011217 ; LMGP01011055 ; LMGP01011201 ; LMGP01011202 ; LMGP01011204 ;LMGP01011206 |
